# In Vitro Evaluation of Iraqi Kurdistan Tomato Accessions Under Drought Stress Conditions Using Polyethylene Glycol-6000

**DOI:** 10.3390/life14111502

**Published:** 2024-11-18

**Authors:** Nawroz Abdul-razzak Tahir, Kamaran Salh Rasul, Djshwar Dhahir Lateef, Rebwar Rafat Aziz, Jalal Omer Ahmed

**Affiliations:** 1 Food Science and Quality Control Department, Bakrajo Technical Institute, Sulaimani Polytechnic University, Sulaymaniyah 46001, Kurdistan Region, Iraq; jalal.ahmed@spu.edu.iq; 2 Horticulture Department, College of Agricultural Engineering Sciences, University of Sulaimani, Sulaymaniyah 46001, Kurdistan Region, Iraq; kamaran.rasul@univsul.edu.iq; 3 Biotechnology and Crop Science Department, College of Agricultural Engineering Sciences, University of Sulaimani, Sulaymaniyah 46001, Kurdistan Region, Iraq; djshwar.lateef@univsul.edu.iq; 4 Gardens Unit, University of Sulaimani, Sulaymaniyah 46001, Kurdistan Region, Iraq; rebwar.aziz@univsul.edu.iq

**Keywords:** *Solanum lycopersicum*, abiotic stress, germination, growth, morphological characteristics, biochemical traits

## Abstract

Drought is one of the major abiotic stresses that affect plant growth and productivity, and plant stress responses are affected by both the intensity of stress and genotype. In Iraqi Kurdistan, tomato plants play a significant role in the country’s economy. Due to climate change, which causes soil moisture to diminish, the crop’s growth and yield have been dropping in recent years. Accordingly, the effects of simulated drought stress on germination parameters were assessed in 64 tomato accessions gathered from the Iraqi Kurdistan region in order to identify sensitive and tolerant accessions. In this respect, the responses associated with drought stress were observed phenotypically and biochemically. Germination percentage (GP) and morphological characteristics such as root length (RL), shoot length (SL), and shoot fresh weight (SFW) were significantly reduced in both stress treatments with polyethylene glycol (PEG-6000) (7.5% PEG and 15% PEG). On the other hand, significant changes in biochemical profiles such as proline content (PC), soluble sugar content (SSC), total phenolic content (TPC), antioxidant activity (AC), guaiacol peroxidase (GPA), catalase (CAT), and lipid peroxidation (LP) in tomato accessions were detected; all biochemical traits were increased in most tomato accessions under the PEG-induced treatments compared to the control treatment (0.0% PEG). Three tomato accessions (AC61 (Raza Pashayi), AC9 (Wrdi Be Tow), and AC63 (Sandra)) were found to be the most tolerant accessions under all drought conditions, whereas the performances of the other tested accessions (AC13 (Braw), AC30 (Yadgar), and AC8 (Israili)) were inferior. The OMIC analysis identified the biomarker parameters for differentiating the highly, moderately, and low tolerant groups as PC, SSC, and TPC. This study shows that early PEG-6000 screening for drought stress may help in choosing a genotype that is suitable for growth in water-stressed environments. Hence, Raza Pashayi, Wrdi Be Tow, and Sandra accessions, which had great performances under drought conditions, can be candidates for selection in a breeding program to improve the growth of plants and production in the areas that face water limits.

## 1. Introduction

Tomatoes (*Solanum lycopersicum* L.) are cultivated around the world and have become an economically important crop [1]. Besides that, this plant serves as a model for the introduction of agronomically significant genes into dicotyledonous crop plants [2]. Tomatoes are one of the most important vegetable crops in the horticulture industry today, and they are grown all over the world for fresh consumption or processing. It is the world’s leading vegetable product, accounting for roughly 14% of global production [3]. According to FAOSTAT [4], tomato production in the world in the year 2022 was about 186 million tonnes. The tomato is regarded as a protective food due to its high nutritive value, which includes carotenoids (mainly lycopene and *β*-carotene), ascorbic acid (vitamin C), tocopherols (vitamin E), and polyphenolic compounds (phenols, flavonoids, and phenolic acids) [5]. Tomato consumption reduces risks of inflammatory processes, cancer, and chronic noncommunicable diseases, such as cardiovascular disease, hypertension, diabetes, and obesity [6].

Climate change is regarded as one of the most significant challenges that communities face globally [7]. Climate change causes global warming, water resource scarcity, decreased agricultural production, and food security, impacting crop production and water availability [8,9]. Crop stress represents a harmful deviation from normal physiological processes that cause crop yields to decline. It could be the result of both biotic and abiotic stress [10]. Drought is a significant abiotic stress that has an impact on crop production in tropical, arid, and semi-arid regions [11,12]. Prolonged water deficit conditions in any region result in a drought-like situation that has a significant impact on the affected area’s agricultural productivity [13]. Drought occurs when environmental temperatures are high and soil moisture, as well as relative humidity, are low [14]. All terrestrial plants are susceptible to short-/long-term water deficit conditions, which cause water loss and a decrease in water potential, resulting in a decrease in cell turgor. This has an effect on overall plant growth, development, and fertility [12,15]. Low soil water availability impacts the plant’s internal water content, impairing its physiological and biochemical functions [16]. When plants undergo abiotic stress, they react by producing reactive oxygen species (ROS), which harm different parts of their cells through oxidation [17]. Plants can detoxify reactive oxygen species (ROS) through enzymatic and non-enzymatic adaptation processes, such as the production of chemicals and the activation of antioxidant enzymes [18,19]. This mechanism usually involves using regular antioxidants to scavenge free radicals. ROS production is governed by enzymatic antioxidant defense systems such as ascorbate peroxidase (APX), catalase (CAT), proline oxidase (POX), superoxide dismutase (SOD), monodehydroascorbate reductase, and many others, whereas non-enzymatic catalysts encompass ascorbate, glutathione, carotenoids, proline, phenolic compounds, sugars, and glycine betaine [20,21,22]. In reaction to water constraint, plants accumulate proline, a defensive compound. Due to its abundance in proteins, proline contributes to the integrity of cell membranes and the stability of proteins, hence facilitating the normal functioning of all biological processes. This cyclic amino acid has been extensively studied for its dual functions as an osmolyte, which reduces water loss, and its impact on plant vitality [23]. Monosaccharides and disaccharides, the two primary categories of soluble sugars, are vital for the optimal functioning of all living cells. Soluble sugars, as osmotic molecules, may affect plant water transport and enhance drought resistance. Sugars in solution fulfill several functions, such as regulating osmotic pressure, preserving carbon, neutralizing reactive oxygen species, safeguarding DNA structures, stabilizing proteins, and maintaining membrane integrity. In circumstances of severe dehydration, carbohydrates are essential for protein hydration, surpassing the significance of proline [24,25]. Aromatic compounds containing one or more hydroxyl groups are referred to as phenolics or polyphenols, which are predominantly synthesized by plants as a defense mechanism against stresses. To adapt to their constantly changing environments, plants have evolved to produce thousands of unique phenolic compounds. To withstand detrimental environmental variables such as drought stress, plants accumulate phenolic chemicals within their tissues. These chemicals are crucial for the regulation of plant growth. Phenolic chemicals, as antioxidants, are believed to initiate protective mechanisms against reactive oxygen species (ROS) by reducing the generation of harmful oxidants such as free radicals [26].

Despite its economic importance, the tomato is susceptible to drought stress, particularly during the seedling growth, blooming, and fruit enlargement phases [27,28], which inhibits seed germination, slows plant development, and reduces fruit yield [29]. Abiotic stresses have a negative impact on tomato production, productivity, and quality. Drought, extreme temperature, and high salinity are all abiotic stresses that affect almost every stage of the tomato life cycle. Abiotic stress causes approximately 70% tomato yield loss depending on the plant stage and duration of the stress [2]. Drought tolerance is a developmentally regulated, stage-specific phenomenon that is affected by genotype and the severity of the stress applied. As a result, drought tolerance at one stage of plant development may not be related to the adult stage; thus, the seedling stage has been reported as more sensitive to water stress than total germination in tomato [30]. One important strategy for overcoming this problem is the development of water stress-tolerant cultivars through screening and selection [31]. This study’s objectives are as follows: (1) to use phenotypic analysis to assess the drought tolerance of sixty-four tomato accessions collected in the Iraqi Kurdistan region; (2) to investigate the biochemical responses; (3) to choose the accession that performs the best under drought conditions; and (4) to ascertain the correlation between the biochemical response and the level of tolerance.

## 2. Materials and Methods

### 2.1. Plant Materials

The seeds of 64 tomato accessions were gathered during summer 2019, from six provinces of the Iraqi Kurdistan region: Sulaymaniyah, Erbil, Duhok, Garmian, Raparin, and Halabja. The details of all tomato accessions were described in our previous study by Rasul et al. [32].

### 2.2. Germination Test and Morphological Traits

To assess drought tolerance during germination and seedling growth, polyethylene glycol-MW 6000 (PEG-6000) was used. The tomato seeds were sterilized by soaking them for 6 min in a 4% sodium hypochlorite (bleach) solution, followed by seven washes with distilled water. A disposable plastic petri dish (9 cm in diameter) and two filter papers were used. Each petri dish contained twenty-five seeds from each accession, and five replications were carried out for each treatment. Each petri dish received 10 mL of distilled water, 7.5% PEG, and 15% PEG as the control (T0), treatment 1 (T1), and treatment 2 (T2), respectively. All samples were incubated in an incubator (Daihan LabTech Co., Ltd., Gyeonggi, Republic of Korea) and kept at a temperature of 23 ± 2 °C. Germinated seeds were considered seeds with radical lengths of 2 mm or more to estimate the germination percentage (GP). The seedlings were taken out of the petri dishes after 14 days of growth to evaluate the morphological characteristics: root length (RL), shoot length (SL), fresh weight (FW), and dry weight (DW). The GP was calculated using the following equation:

$$\mathrm{Germination}\left(\%\right)=\frac{\mathrm{Number}\;\mathrm{of}\;\mathrm{germinated}\;\mathrm{seeds}}{\mathrm{Total}\;\mathrm{number}\;\mathrm{of}\;\mathrm{used}\;\mathrm{seeds}}\times 10$$

Following that, all samples were collected and powdered with liquid nitrogen before being stored at −20 °C and used for biochemical tests.

### 2.3. Biochemical Tests

The fresh seedlings of all tomato accessions were ground with liquid nitrogen and used to estimate the following biochemical parameters: proline content (PC in μg/g seedling fresh weight), total phenolic content (TPC in μg gallic acid/g seedling fresh weight), antioxidant activity (AC in μg Trolox/g seedling fresh weight), soluble sugar content (SSC in μg glucose/g seedling fresh weight), catalase activity (CAT in units/min/g seedling fresh weight), guaiacol peroxidase activity (GPA in units/min/g seedling fresh weight) and lipid peroxidation (LP in nmol/g seedling fresh weight), and the methods of the determination for these biochemicals are described in our previous works [33,34,35,36].

### 2.4. Statistical Data Analysis

One-way ANOVA-CRD and Duncan’s new multiple range tests were used in XLSTAT software version 2022.3.1 (Addinsoft, New York, NY, USA) to evaluate significant differences (*p* ≤ 0.01 and *p* ≤ 0.001) among tomato accessions. The box chart and principal component analysis plot were created with XLSTAT software version 2022.3.1, to facilitate the identification and selection of stable, high-yielding, drought-resistant genotypes. The ranking method was employed to determine the optimal accessions based on the specified strategy, using several computed traits. As selected criteria for the best accessions across all traits, the average number of ranks (ASRs) and the stress tolerance index (STI) were developed [37]. These metrics are valuable for identifying tolerant genotypes based on their productivity in adverse situations. The omics analysis was conducted by XLSTAT software version 2022.3.1.

## 3. Results

### 3.1. Morphological Trait Performance Under Drought Stress Conditions

**Figure 1:**
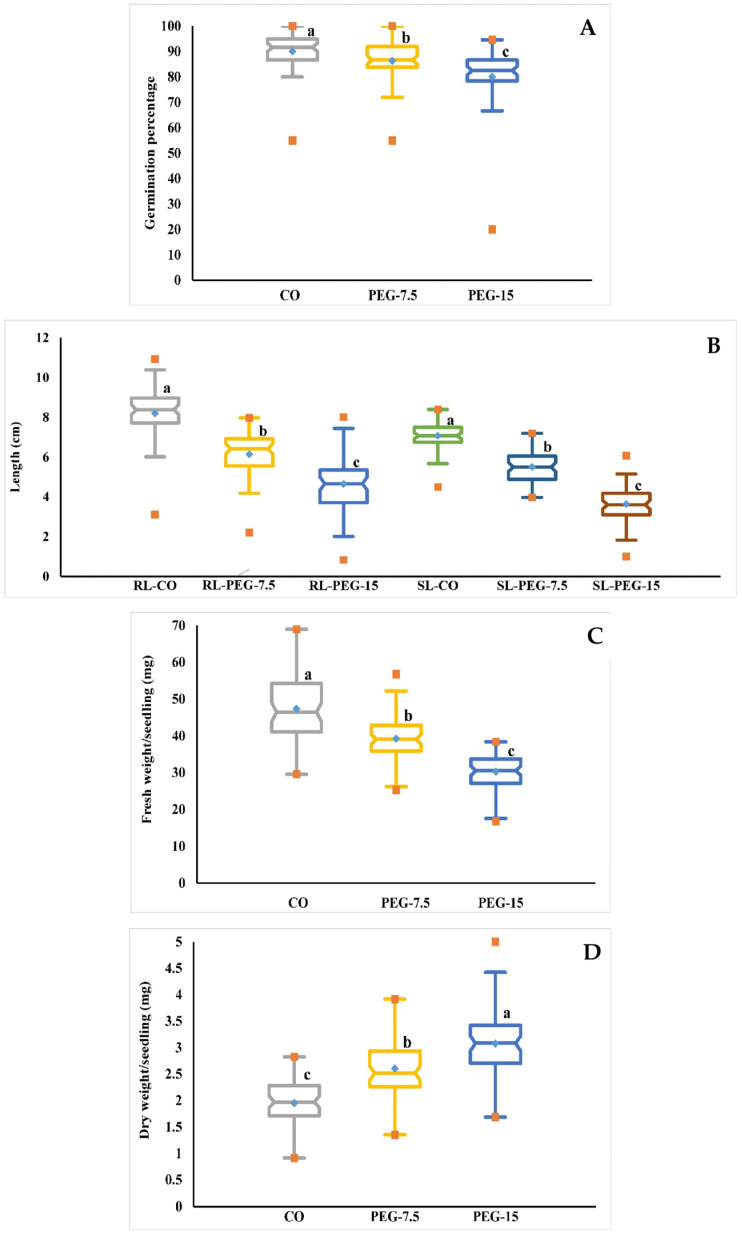
A box plot comparing the phenotypic characteristics of plants grown in control and stress conditions. (**A**) Germination percentage (GP), (**B**) root length (RL) and shoot length (SL), (**C**) seedling fresh weight (FW), and (**D**) seedling dry weight (DW). The values provided represent an average of the results from the three separate measurements. Control (CO or T0 = 0.00% PEG) and PEG concentrations: T1 = 7.5% PEG and T2 = 15% PEG. Different letters represent a significant difference between the mean values according to Duncan’s Multiple-Range Test (*p* ≤ 0.01). A blue dot in the box indicates the mean, while orange dots represent the minimum and maximum values.

In this study, tomato accessions were cultivated in control medium (T0 or 0% PEG) and under drought-induced (PEG treatment) conditions (T1 (7.5% PEG) and T2 (15% PEG)) to evaluate the effects of drought on the tested tomato accessions in terms of growth traits, mainly germination percentage (GP in %), root length (RL in cm), shoot length (SL in cm), fresh weight of seedling (FW in mg), and dry weight of seedling (DW in mg). According to the ANOVA analysis and box charts computed from the data of 64 accessions presented in the supplemental file, there was a significant difference between the tomato accessions under both control and drought-induced (PEG treatment) conditions (Table 1 and Figure 1). Table 2 shows that for all morphological traits (GP, RL, SL, FW, and DW), there were highly significant differences between accessions, PEG concentrations, and interactions between them (*p* ≤ 0.01), based on the ANOVA analysis performed on the data of 64 accessions reported in the supplementary file. All morphological characteristics except DW decreased significantly as PEG concentration increased for all accessions (Table 1). In the analysis of GP under control conditions, the performance of this trait ranged between 55% (AC13) and 100% (AC4, AC6) under the control condition, with a mean of 90.10%, while RL ranged from 3.12 (AC13) to 10.93 (AC29) cm, with an average of 8.21 cm, and SL ranged from 4.50 (AC1) to 8.41 (AC63) cm, with an average of 7.08 cm. FW ranged from 29.55 (AC11) to 68.97 (AC20) mg, with a mean of 47.35 mg, and DW ranged from 0.92 (AC11) to 2.83 (AC20) mg, with a mean of 1.95 mg (Table 1 and Table S1).

The mean values of GP in T1 (7.5% PEG) stress ranged from 55% (AC13) to 100% (AC4, AC6), with an average of 86.34%, and RL ranged from 2.2 (AC13) to 7.98 (AC54) cm, with an average of 6.14 cm. SL ranged from 3.98 (AC18) to 7.20 (AC43) cm with an average of 5.52 cm; FW ranged from 25.31 (AC18) to 56.78 (AC10) mg with an average of 39.24 mg; and DW ranged from 1.35 (AC42) to 3.92 (AC10) mg with an average of 2.61 mg (Table 1 and Table S2).

The mean values of the accessions varied between 20.00% (AC13) and 94.67% (AC61) with an average of 80.04%, 0.83 (AC13) to 8.02 (AC56) cm with a mean of 4.65 cm, 1.00 (AC13) to 6.07 (AC63) cm with an average of 3.63 cm, 16.72 (AC5) to 38.39 (AC60) mg with an average of 30.25 mg, and 1.69 (AC42) to 5.00 (AC42) mg with a mean of 3.08 mg for GP, RL, SL, FW, and DW, respectively, under induced drought stress T2 (15% PEG) (Table 1 and Table S3). The box charts (Figure 1) of all traits show significant variations between T0 (control), T1 (7.5% PEG), and T2 (15% PEG). All tomato accessions under the control condition had significantly higher trait values when compared to stressed plants, except the DW trait value, which was lower compared to T1 (7.5% PEG) and T2 (15% PEG). These findings indicate that T2 (15% PEG) was more effective at reducing seedling growth.

The mean value of all morphological traits under all drought conditions showed a highly significant variation among all accessions (Table S4). AC6 and AC13 had the maximum and minimum GP values, which were 97.22% and 43.33%, respectively, while AC50 and AC8 had the longest and shortest root length (RL) values by 8.09 cm and 4.05 cm, respectively. AC63 (7.10 cm) and AC37 (4.33 cm) had the longest and shortest shoot lengths (SL), respectively. The highest values of FW and DW are indicated by AC30 and AC8, with 53.16 mg and 3.71 mg, respectively, while the lowest values of these characters were revealed in AC6 with 27.82 mg and AC11 with 1.38 mg.

The interaction values between tomato accessions and PEG induced are shown in Table S5, and a highly significant difference between them has been revealed. The combination of AC4 + control and AC6 + Control recorded the highest values of germination percentages (GP), at 100%, while the lowest value was revealed for AC13 + PEG-15, with 20%. The longest root length (RL) and shoot length (SL) were recorded for the interaction of AC29 + control and AC63 + control, which were 10.93 cm and 8.41cm, respectively, while the shortest values of these traits were indicated under the combination of AC13 + PEG-15, at 0.83 cm and 1.00 cm, respectively. The highest and lowest values of FW were revealed under the interaction of AC20 + control and AC5 + PEG-15, which were 68.97 mg and 16.72 mg, respectively. The interaction of AC11 + control indicated the lowest value of DW by 0.92 mg, whereas the combination of AC9 + PEG-15 recorded the highest value of DW, which was 5.00 mg.

### 3.2. Biochemical Markers Assays

In this investigation, seven biochemical markers, including proline content (PC), soluble sugar content (SSC), total phenolic content (TPC), antioxidant activity (AC), catalase (CAT), guaiacol peroxidase activity (GPA), and lipid peroxidation (LP), were assessed to detect the response of tested tomato accessions to different concentrations of PEG.

**Figure 2:**
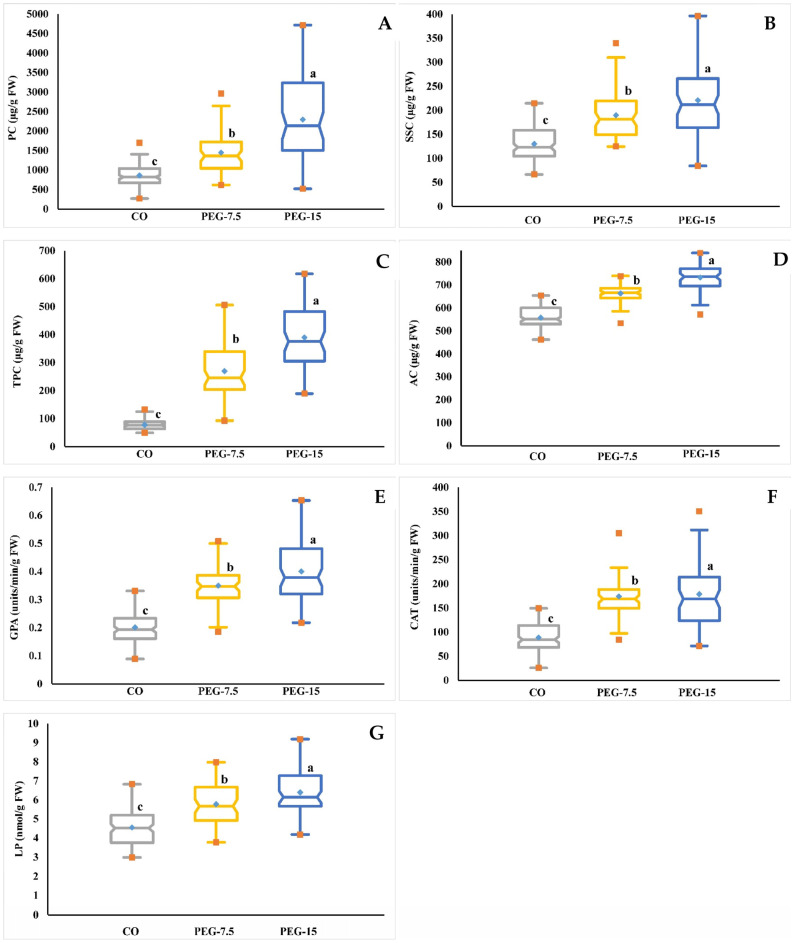
The box charts depict the differentiation of biochemical traits in tomato seedlings under normal and drought stress conditions. (**A**) proline content (PC), (**B**) soluble sugar content (SSC), (**C**) total phenolic content (TPC), (**D**) antioxidant activity (AC), (**E**) guaiacol peroxidase activity (GPA), (**F**) catalase activity (CAT), and (**G**) lipid peroxidation (LP) assay in tomato accessions subjected to control and drought stress. The values given are the averages of the three measurements for control (CO) and PEG concentrations (T1 = 7.5% PEG and T2 = 15% PEG). According to Duncan’s Multiple-Range Test (*p* ≤ 0.01), different letters represent a significant difference between the mean values. A blue dot in the box represents the mean, while red dots represent the minimum and maximum values.

The ANOVA analysis (Table 1) and box charts (Figure 2) reveal significant differences in the biochemical results of PC, SSC, TPC, AC, GPA, CAT, and LP from the control (T0) and both PEG treatments (T1, 7.5% PEG and T2, 15% PEG) performed under drought conditions. Moreover, all biochemical traits were significantly different between accessions, PEG concentrations, and their interactions (*p* ≤ 0.001) (Table 2).

All biochemical traits increased with an increase in induced PEG concentrations, and stressed tomato seedlings produced more of these chemical compounds than control tomato seedlings (Table 1). To evaluate the tomato’s response to PEG’s low water potential, the proline content (PC) and soluble sugar content (SSC) were measured. Proline content (PC) progressively increased as the PEG concentration increased. The mean of all accessions under the control condition (0.0% PEG) was 854.45 µg/g FW, and PC was increased significantly under T1 (7.5% PEG) and T2 (15% PEG), which were 1447.34 µg/g FW and 2295.87 µg/g FW, respectively (Table 1). AC1 and AC42 showed the minimum and maximum PCs under control conditions, which were 268.87 µg/g FW and 1697.59 µg/g FW, respectively (Table S6), while AC61 (2961.18 µg/g FW) (S7) and AC27 (4714.77 µg/g FW) (S8) showed the highest PC values under T1 (7.5% PEG) and T2 (15% PEG), respectively.

AC11 (Table S7) and AC30 (Table S8) had the lowest values of this chemical trait under both induced PEG treatments, with 617.08 µg/g FW and 520.15 µg/g FW, respectively. These findings show that T2 (15% PEG) was more effective on sensitive accessions than tolerant accessions. Under T0 (control), T1 (7.5% PEG), and T2 (15% PEG), the average values of soluble sugar contents (SSC) were 129.97 µg/g FW, 189.86 µg/g FW, and 220.88 µg/g FW, respectively. The highest and lowest values of SSC under control conditions were 66.42 µg/g FW and 214.57 µg/g FW; at T1 (7.5% PEG) they were 124.75 µg/g FW and 339.88 µg/g FW; and at T2 (15% PEG) they were 84.32 µg/g FW and 396.36 µg/g FW, respectively (Table 1 and Tables S6–S8).

Drought stress affected total phenolic content (TPC) and antioxidant activity (AC) in seedlings of all tomato accessions using PEG concentrations compared to control conditions. By increasing the induced PEG concentrations, the average values of these chemical parameters were increased (Table 1). The averages of TPC and AC were 78.33 µg/g FW and 557.82 µg/g FW under the control condition, respectively, while AC28 and AC60 had the lowest values of these traits, at 49.96 µg/g FW and 461.89 µg/g FW, respectively, and the maximum values were shown by AC6 and AC11 with 131.80 µg/g FW and 653.78 µg/g FW, respectively (Table 1 and Table S6). The mean values of TPC and AC were 269.15 µg/g FW and 663.47 µg/g FW, respectively, while 7.5% PEG was induced. The lowest and highest values of TPC under T1 (7.5% PEG) were recorded by AC11, with 92.85 µg/g FW, and AC61, with 505.58 µg/g FW, respectively. The minimum and maximum values of AC at T1 (7.5% PEG) were indicated by AC14 with 534.19 µg/g FW and AC24 with 738.92 µg/g FW, respectively (Table 1 and Table S7). When compared to the control condition, the T2 (15% PEG) treatment yielded the highest TPC and AC values, with TPC averaging 389.86 µg/g FW and AC averaging 732.29 µg/g FW. The minimum values of TPC and AC for this treatment were indicated by AC11 with 189.10 µg/g FW and AC14 with 570.68 µg/g FW, respectively, while the maximum values of TPC and AC were shown in AC61, with 617.19 µg/g FW, and AC6, with 839.59 µg/g FW, respectively (Table 1 and Table S8).

The enzymatic parameters of guaiacol peroxidase activity (GPA), catalase activity (CAT), and lipid peroxidation (LP) of tomato seedlings were increasingly impacted by boosted drought stress. The GPA mean values under T0 (Control), T1 (7.5% PEG), and T2 (15% PEG) conditions were 0.20, 0.35, and 0.40 units/min/g FW, respectively, whereas the CAT means were 88.88, 174.21, and 178.77 units/min/g FW, respectively, and the average values of LP were 4.55, 5.78, and 6.39 nmol/g FW, respectively (Table 1). The minimum values of GPA at T0 (AC9 and AC19), T1 (AC8), and T2 (AC19) were 0.089, 0.189, and 0.218 units/min/g FW, respectively, and the minimum values of CAT were 25.97, 84.42, and 71.43 units/min/g FW in AC2, AC2, and AC13, respectively. For LP, values of 2.98, 3.77, and 4.18 nmol/g FW were given under AC18, AC27, and AC27, respectively (Table 1 and Tables S6–S8). The maximum value of GPA under control conditions was noted in AC50 and AC54, which was 0.331 units/min/g FW, and the maximum value of CAT was seen in AC6, AC14, AC20, AC29, AC31, and AC59, with 149.35 units/min/g FW, while the value of LP was 6.82 nmol/g FW in AC5. The maximum values of GPA, CAT, and LP were recorded in AC50, AC61, and AC45, with 0.508 units/min/g FW, 305.19 units/min/g FW, and 7.97 nmol/g FW, respectively, in T1 (7.5% PEG). Using T2 (15% PEG), the highest values of GPA, CAT, and LP were 0.654 units/min/g FW, 350.65 units/min/g FW, and 9.19 nmol/g FW in AC54, AC61, and AC5, respectively.

The average values of all biochemical traits under drought conditions (T0, T1, and T2) are shown in Table S9. The data attained reveal the highest significant differences among all accessions. AC39 and AC11 showed the maximum and the minimum PC values, with 2786.48 µg/g FW and 641.61 µg/g FW, respectively. The highest and lowest SSC values were given by AC58 and AC57, at 297.18 µg/g FW and 95.53 µg/g FW and 403.83 µg/g FW and 114.07 µg/g FW, respectively. AC61 and AC11 had the highest and lowest TPC values. The two accessions with the highest and lowest AC values were AC611 and AC58, with 708.51 µg/g FW and 553.78 µg/g FW, respectively. The maximum values of GPA, CAT, and LP were recorded by AC54, AC61, and AC5, with 0.495 units/min/g FW, 242.42 units/min/g FW, and 7.94 nmol/g FW, respectively, while the minimum values of these traits were revealed by AC19, AC2, and AC27, with 0.175 units/min/g FW, 69.26 units/min/g FW, and 3.78 nmol/g FW, respectively.

The interactions of accessions with drought conditions for all biochemicals are indicated in Table S10. The highest values of PC and SSC were revealed under the combination of AC27 + PEG-15, which were 4714.77 µg/g FW and 396.36 µg/g FW, respectively, while the lowest PC was indicated at the interaction of AC1 + control, with 268.87 µg/g FW, and the minimum SSC was revealed at AC8 + control, being 66.42 µg/g FW. In the case of TPC and AC, the maximum values were recorded by the combinations of AC61 + PEG-15 (617.19 µg/g FW) and AC6 + PEG-15 (839.59 µg/g FW), respectively, while the minimum values were presented by the interaction of AC28 + control and AC60 + control, with 49.96 µg/g FW and 461.89 µg/g FW, respectively. The highest values of GPA, CAT, and LP were 0.654 units/min/g FW, 350.65 units/min/g FW, and 9.19 nmol/g FW, given at the interactions of AC54 + PEG-15, AC61 + PEG-15, and AC5 + PEG-15, respectively; meanwhile, the lowest values were recorded by the combinations of AC9 + control (0.089 units/min/g FW), AC2 + control (25.97 units/min/g FW), and AC18 + control (2.98 nmol/g FW), respectively.

### 3.3. Ranking of Tomato Accessions for Morphological Traits Under Drought Stress Conditions

The ranking technique was carried out under all treatment conditions in accordance with the process established by Ketata et al. [38]. The average rank (AR) and stress tolerance index (STI) were used as indicators for selecting the best tomato accessions. In light of this, the best-performing accession for the traits obtained the lowest AR and heights STI, and was deemed to be tolerant to various concentrations of cadmium heavy metal stress, in contrast to susceptible tomato accessions.

A ranking approach based on germination percentage and seedling growth (root length + shoot length) was used to identify the best accessions with the tested features. The best accessions for germination and seedling growth were determined by using the average sum of rankings (AR) as a predictor. The best drought tolerance accession was chosen based on the highest stress tolerance index (STI) and the lowest average number of ranks (AR).

AC4 and AC63 performed the best under drought stress treatments T1 (7.5% PEG) and T2 (15% PEG), respectively, while AC13 performed the worst under both conditions (Table 3). The combination of T1 and T2 yielded the best tomato accessions with resistance to drought stress. According to the results (Table 3), AC61, AC9, and AC63 had the highest ranking for response to PEG, and it can be assumed that these accessions are the most resistant to drought stress. On the other hand, AC13, AC30, and AC8 were the most susceptible accessions to PEG.

### 3.4. Biochemical Markers Related to Degree of Tolerance by Omics Analysis

In genomics and biochemistry, statistical differential expression (omics approach) was used to identify features that are affected by descriptive variables. Using the mean values of chemical features obtained in response to different induced PEG (Table 4), in our case study, it is possible to determine the level of tolerance and susceptibility.

In this investigation, three biochemical traits (PC, SSC, and TPC) showed highly significant responses of tomato accessions under the T1 (7.5% PEG) and T2 (15% PEG) applications. The mean value of PC under T1 varied between 1642.00 and 821.24 for the high and low tolerance responses, respectively, whereas it varied between 1880.00 and 881.18 for the high and low tolerant responses, respectively, under T2. In the case of SSC, the mean values for high-tolerance response were 204.13 and 196.86 under T1 and T2, respectively, whereas the mean values for low-tolerance response were 142.42 and 143.45 under T1 and T2, respectively. Under T1, the TPC trait recorded mean values for high- and low-tolerance responses of 302.84 and 151.41, respectively, and indicated mean values of 353.89 and 241.08 for high- and low-tolerance responses under T2.

## 4. Discussion

Plant growth is caused by cell division, cell enlargement, and differentiation, and is influenced by a variety of genetic, physiological, ecological, and morphological processes, as well as their interactions [39]. Effective screening to differentiate between drought-tolerant and drought-sensitive genotypes based on easily measurable traits can aid in the investigation of tomato accessions’ stress capacity. Plants’ drought responses are the result of dynamic and diverse adaptations. As a result, they can be studied at multiple levels, from morphological and physiological changes in organs to complex reactions to gene expression and regulation rates, as well as biochemical reactions at the cell and organ levels. Damage to physiological and biochemical processes, such as deferred stomatal conductance, diminished nutrient uptake, breakdown of leaf pigments, reduced photosynthesis, a halt in the rate of net assimilation and photosystem photochemical efficiency parameters, an increase in ROS, and oxidative damage caused by water stress, reduced the morphological features [40]. Abiotic stresses, such as drought, affect cell homeostasis, photophosphorylation, soluble proteins, the presence of proteins in thylakoid membranes, and photosynthetic pigments, all of which slow down photosynthesis [41]. Drought stress has an effect on plant protein content and quality. Plants synthesize massive amounts of ribosomal and chaperone proteins under adverse environmental conditions [42]. All of these proteins are essential for maintaining proper protein production, folding denatured proteins, and the disaggregation of denatured proteins, all of which aid in stress adaptation [43]. Polyethylene glycol (PEG), a synthetic hydrophilic and biocompatible polymer, has a variety of agricultural applications. A molecule’s toxicity is proportional to its molecular weight. Large-molecular-weight polyethylene glycol (PEG) molecules (MW 6000 or more) are regarded as non-toxic and cannot enter plants. Plants absorbed PEGs with molecular weights less than 6000 slowly, and only the physically wounded roots were affected. When low-molecular-weight PEG was collected at the leaf edges, it spread into the mesophyll cells. The leaves withered and died due to the low-molecular-weight PEG, which inhibited transpiration, water potential, and respiration [44].

The first step of drought-induced moisture deficiency is disrupted germination, which results in poor plant standing at the early seedling level and impedes early crop development. Increased PEG concentrations decrease germination percentage and seedling growth due to low hydraulic conductivity and high viscosity, disrupting essential seed imbibition processes and causing water to be unavailable to seeds. Reduced seedling growth is caused by restrictions in cell divisions and enlargement resulting from reduced water absorption [45,46].

Our findings in vitro, derived using different PEG concentrations, revealed that all tomato accessions respond differently to all PEG concentrations for all morphological and biochemical traits. The in vitro experiment revealed natural variations among the studied accessions, which revealed different growth characteristics in the control and stress conditions of the sixty-four tomato accessions, and significant decreases were noted as the concentration of PEG increased (Figure 1 and Tables S1–S5). These findings are consistent with previous research, and provide further evidence of PEG’s suitability as a molecule for simulating droughts in vitro. These findings are consistent with previous research and show that the response to droughts is dependent on both accessions and stress levels [47]. The morphological parameters GP, RL, SL, and FW decreased significantly as the PEG concentration increased. The GP value was 90.1% at T0 (0% PEG), but decreased to 86.34% and 80.04% at T1 (7.5% PEG) and T2 (15% PEG), respectively. Under T0, T1, and T2, the RL values were 8.21, 6.14, and 4.65 cm, respectively. These results are similar with the finding given by Zhou et al. [48], where the DW was increased. This result could be attributed to the accumulation of sugar and PEG molecules within the plant cell, which were exposed to drought via PEG [49]. In response to drought stress, plants produce and store the necessary solutes, such as amino acids, polyols, and carbohydrates, to support osmotic balance and water absorption and retention [26]. The biochemical traits PC, SSC, TPC, AC, GPA, CAT, and LP were gradually increased, while the PEG concentration increased, particularly in the tolerant accessions (Figure 2 and Tables S6–S10). For example, the PC values were 854.45, 1447.35, and 2295.87 µg/g FW, and SSC values were 129.97, 189.86, and 220.88 µg/g FW under T0, T1, and T2, respectively. One of the major causes of reduced development under stress conditions is oxidative stress. The buildup of certain chemical molecules, particularly those with allelochemical characteristics, at higher quantities inhibits plant growth [50]. SSC has several roles, including osmotic modification, carbon preservation, the detoxification of ROS, the defense of membrane integrity, the safety of DNA structures, and protein stabilization. In serious dehydrated conditions, sugars become an important water substitute, perhaps more than proline, for the hydration of proteins [51]. Proline is osmotic and plays a significant role in the stabilization of the membrane. It also works by scavenging free radicals and syncing the redox ability of the cells, which allows the plants to fight abiotic stress [52]. Prolonged stress, on the other hand, may cause ROS aggregation at the plasma membrane and, as a result, cause cell damage. To reduce ROS production, the plant must increase the activity of antioxidant/detoxifying systems such as APX, SOD, CAT, and POD [53,54].

TPC was found to be significantly higher in T1 and T2 than under normal conditions in this study. Several studies have found that extreme drought stress increases antioxidant activity and polyphenol and flavonoid levels. The bioactivity of phenolic molecules is regarded as a signaling mechanism that leads to protective mechanisms against ROS invasion by serving as an antioxidant agent to reduce the generation of cell-damaging oxidants such as free radicals [55,56]. Peroxidases are enzymes that are important for plant development and abiotic stress response [57]. They are involved in various physiological processes, including lignin biosynthesis, cell wall remodeling, and plant defense against pathogens [58]. Peroxidase was down-regulated in response to drought stress, which could indicate a reduction in the capacity of a tolerant accession to detoxify ROS, such as H_2_O_2_, leading to ROS accumulation. Damage to plants from oxidative stress can be measured in a number of ways, one of which is lipid peroxidation. In most cases, cell death occurs when membrane lipid peroxidation weakens the membranes, allowing minerals and other vital cellular compounds to flow out [59]. The omics analysis identified PC, SSC, and TPC as three biomarkers associated with the degree of tolerance among all studied biochemical traits. These biomarkers enabled the differentiation of genotypes with high and low tolerance [60].

In order to test for drought tolerance in different accessions, a potential germplasm must be selected. The study’s findings show that different tomato accessions responded differently in genetic terms to drought stress. From this vantage point, creating new varieties that are well-suited to areas with scarce water resources involves selecting the most drought-resistant accessions and then using them as parents in a cross-breeding procedure.

## 5. Conclusions

Natural stress tolerance is a complicated phenomenon that includes numerous physiological and biochemical processes. As a reflective basis for drought tolerance of selected accessions, germination and seedling growth were assessed under induced osmotic stress mediated by PEG treatments. Seed germination and seedling growth were significantly reduced by all PEG concentrations. The results of this study show that the accessions of AC61 (Raza Pashayi), AC9 (Wrdi Be Tow), and AC63 (Sandra) seemed to be the most tolerant, while the AC13 (Braw), AC30 (Yadgar), and AC8 (Israili) accessions were most susceptible to PEG stress. The tolerant accessions have expressed the smallest decrease in seed germination and seedling growth traits, although there is a significant increase in biochemical traits except for LP. According to the omics analysis, PC, SSC, and TPC are the biomarker characteristics that allow a differentiation between high and low tolerance genotypes. Finally, germination and seedling growth characteristics offer a short-term, low-cost method of rapidly determining tomato accessions that are sensitive or tolerant. So, to further understand the roles of various metabolites in adaptation to harsh environments, it is recommended that future studies examine metabolic modifications (secondary metabolites and proteomic profiles) in tomato accessions with varying degrees of stress tolerance. In a future project, we will use the most effective and least efficient genotypes from in vitro experiments under various water stress regimes in greenhouse conditions to determine the relationship between the results of in vitro studies and greenhouse experiments.

## Figures and Tables

**Table 1:** The descriptive statistics of morpho-chemical traits computed from the data of 64 accessions stated in the supplementary file under control conditions and different PEG concentrations.

Traits	T0 (Control or 0% PEG)				
	Min	Max	Mean	F	Pr > F
GP	55.00	100.00	90.10	12.20 ***	<0.0001
RL	3.12	10.93	8.21	15.10 ***	<0.0001
SL	4.50	8.41	7.08	5.62 ***	<0.0001
FW	29.55	68.97	47.35	7.14 ***	<0.0001
DW	0.92	2.83	1.95	6.68 ***	<0.0001
PC	268.87	1697.59	854.45	104.83 ***	<0.0001
SSC	66.42	214.57	129.97	47.89 ***	<0.0001
TPC	49.96	131.8	78.33	40.42 ***	<0.0001
AC	461.89	653.78	557.82	89.32 ***	<0.0001
GPA	0.09	0.33	0.20	6.64 ***	<0.0001
CAT	25.97	149.35	88.88	8.06 ***	<0.0001
LP	2.98	6.82	4.55	110.74 ***	<0.0001
	**T1 (7.5% PEG)**				
GP	55.00	100.00	86.34	8.24 ***	<0.0001
RL	2.21	7.98	6.14	11.00 ***	<0.0001
SL	3.98	7.20	5.52	7.02 ***	<0.0001
FW	25.31	56.78	39.24	6.64 ***	<0.0001
DW	1.35	3.92	2.61	6.58 ***	<0.0001
PC	617.08	2961.18	1447.35	677.37 ***	<0.0001
SSC	124.75	339.88	189.86	515.62 ***	<0.0001
TPC	92.85	505.58	269.15	2522.69 ***	<0.0001
AC	534.19	738.92	663.47	474.26 ***	<0.0001
GPA	0.19	0.51	0.35	41.63 ***	<0.0001
CAT	84.42	305.19	174.21	52.79 ***	<0.0001
LP	3.77	7.97	5.78	693.51 ***	<0.0001
	**T2 (15% PEG)**				
GP	20.00	94.67	80.04	11.84 ***	<0.0001
RL	0.83	8.02	4.65	17.23 ***	<0.0001
SL	1.00	6.07	3.63	9.79 ***	<0.0001
FW	16.72	38.39	30.25	7.63 ***	<0.0001
DW	1.69	5.00	3.08	8.09 ***	<0.0001
PC	520.15	4714.77	2295.87	4439.84 ***	<0.0001
SSC	84.32	396.36	220.88	1334.72 ***	<0.0001
TPC	189.10	617.19	389.86	6249.08 ***	<0.0001
AC	570.68	839.59	732.29	105.95 ***	<0.0001
GPA	0.22	0.65	0.40	163.68 ***	<0.0001
CAT	71.43	350.65	178.77	115.61 ***	<0.0001
LP	4.18	9.19	6.39	524.02 ***	<0.0001

GP, germination percentage (%); RL, root length (cm); SL, shoot length (cm); FW, fresh weight (g); DW, dry weight (g); PC, proline content (µg/g FW); SSC, soluble sugar content (µg/g FW); TPC, total phenolic content (µg/g FW); AC, antioxidant activity (µg/g FW); GPA, guaiacol peroxidase activity (units/min/g FW); CAT, catalase (units/min/g FW), and LP, lipid peroxidation (nmol/g FW). ***, very highly significant at level *p* ≤ 0.001; Pr, probability.

**Table 2:** Mean square and probability values of accessions, PEG concentrations, and their combination on the morphological and biochemical parameters obtained using the data of 64 accessions specified in the supplementary file.

Traits	Mean Square of Accessions	Pr > F	Mean Square of PEG Concentration	Pr > F	Mean Square of Accessions × PEG Concentration	Pr > F
GP	26.51 ***	<0.0001	184.31 ***	<0.0001	2.87 ***	<0.0001
RL	29.84 ***	<0.0001	1717.21 ***	<0.0001	6.57 ***	<0.0001
SL	12.96 ***	<0.0001	2319.67 ***	<0.0001	4.64 ***	<0.0001
FW	14.82 ***	<0.0001	715.98 ***	<0.0001	3.21 ***	<0.0001
DW	16.30 ***	<0.0001	493.62 ***	<0.0001	2.75 ***	<0.0001
PC	3091.76 ***	<0.0001	111,025.50 ***	<0.0001	1050.77 ***	<0.0001
SSC	1121.39 ***	<0.0001	25,652.06 ***	<0.0001	311.35 ***	<0.0001
TPC	5368.06 ***	<0.0001	631,700.60 ***	<0.0001	1434.61 ***	<0.0001
AC	239.4224 ***	<0.0001	36,960.56 ***	<0.0001	135.4114 ***	<0.0001
GPA	133.43 ***	<0.0001	6638.74 ***	<0.0001	33.83 ***	<0.0001
CAT	112.90 ***	<0.0001	4799.69 ***	<0.0001	49.45 ***	<0.0001
LP	1392.67 ***	<0.0001	28,019.48 ***	<0.0001	114.31 ***	<0.0001

GP, germination percentage (%); RL, root length (cm); SL, shoot length (cm); FW, fresh weight (g); DW, dry weight (g); PC, proline content (µg/g FW); SSC, soluble sugar content (µg/g FW); TPC, total phenolic content (µg/g FW); AC, antioxidant activity (µg/g FW); GPA, guaiacol peroxidase activity (units/min/g FW); CAT, catalase (units/min/g FW), and LP, lipid peroxidation (nmol/g FW). ***, very highly significant at level *p* ≤ 0.001; Pr, probability.

**Table 3:** Rankings of sixty-four tomato accessions using the stress tolerance index (STI) and average number of ranks (AR) under the drought conditions of T1 (7.5% PEG), T2 (15% PEG), and both treatments (T1 and T2). The best accessions had the highest STI and lowest AR, and the lowest rank was assigned to the accession with the most consistent performance.

	T1 (7.5% PEG)				T2 (15% PEG)				T1 + T2		
Accession Code	STI	AR	Rank	Accession Code	STI	AR	Rank	Accession Code	STI	AR	Rank
AC4	1.15	4.64	1.00	AC63	1.08	5.27	1.00	AC61	1.10	2.73	1.00
AC6	1.14	5.91	2.00	AC61	1.07	3.91	2.00	AC9	1.06	7.09	2.00
AC61	1.13	3.00	3.00	AC6	1.01	14.64	3.00	AC63	1.09	7.36	3.00
AC43	1.12	13.55	4.00	AC9	1.00	9.73	4.00	AC6	1.08	10.00	4.00
AC63	1.11	18.82	5.00	AC39	1.00	12.00	5.00	AC54	0.98	10.09	5.00
AC9	1.11	5.55	6.00	AC43	0.99	19.18	6.00	AC39	1.04	12.09	6.00
AC42	1.11	9.36	7.00	AC64	0.99	24.00	7.00	AC27	0.97	13.91	7.00
AC64	1.09	22.91	8.00	AC15	0.98	15.82	8.00	AC4	1.07	14.45	8.00
AC39	1.08	18.55	9.00	AC54	0.98	7.82	9.00	AC26	1.03	14.82	9.00
AC60	1.08	20.82	10.00	AC26	0.98	16.64	10.00	AC43	1.05	14.91	10.00
AC41	1.07	25.55	11.00	AC4	0.98	25.00	11.00	AC38	0.95	16.91	11.00
AC26	1.07	18.18	12.00	AC41	0.97	26.91	12.00	AC42	1.03	17.36	12.00
AC44	1.05	20.45	13.00	AC58	0.96	15.55	13.00	AC31	0.97	17.55	13.00
AC48	1.05	11.00	14.00	AC50	0.95	12.64	14.00	AC50	0.97	18.09	14.00
AC47	1.04	9.91	15.00	AC42	0.95	26.73	15.00	AC32	0.98	19.45	15.00
AC17	1.03	31.45	16.00	AC27	0.95	13.00	16.00	AC47	0.95	20.00	16.00
AC32	1.02	24.91	17.00	AC32	0.94	21.55	17.00	AC28	0.97	22.00	17.00
AC46	1.02	15.00	18.00	AC29	0.94	24.82	18.00	AC46	0.96	22.36	18.00
AC29	1.02	29.09	19.00	AC1	0.94	14.27	19.00	AC64	1.04	22.45	19.00
AC34	1.01	29.09	20.00	AC31	0.93	18.00	20.00	AC55	0.89	22.82	20.00
AC7	1.01	19.73	21.00	AC28	0.93	21.27	21.00	AC41	1.02	25.27	21.00
AC27	1.00	16.55	22.00	AC34	0.93	25.55	22.00	AC12	0.92	25.36	22.00
AC28	1.00	29.18	23.00	AC17	0.92	34.45	23.00	AC29	0.98	25.45	23.00
AC62	1.00	20.55	24.00	AC38	0.92	16.27	24.00	AC34	0.97	25.45	24.00
AC31	1.00	23.27	25.00	AC60	0.90	37.36	25.00	AC56	0.88	26.09	25.00
AC54	0.98	19.82	26.00	AC12	0.90	20.18	26.00	AC58	0.96	26.55	26.00
AC50	0.98	32.18	27.00	AC2	0.90	32.82	27.00	AC62	0.94	26.55	27.00
AC15	0.98	39.82	28.00	AC55	0.90	16.27	28.00	AC53	0.87	27.82	28.00
AC38	0.97	20.36	29.00	AC46	0.90	29.55	29.00	AC15	0.98	28.27	29.00
AC58	0.97	38.91	30.00	AC23	0.89	27.82	30.00	AC60	0.99	28.73	30.00
AC36	0.97	38.55	31.00	AC25	0.89	34.18	31.00	AC24	0.89	28.82	31.00
AC40	0.95	36.55	32.00	AC36	0.89	36.73	32.00	AC7	0.94	31.27	32.00
AC25	0.95	41.82	33.00	AC62	0.88	32.64	33.00	AC45	0.85	31.36	33.00
AC22	0.95	32.27	34.00	AC56	0.88	20.64	34.00	AC17	0.98	32.09	34.00
AC16	0.94	33.55	35.00	AC53	0.88	21.82	35.00	AC48	0.94	33.00	35.00
AC12	0.94	35.36	36.00	AC18	0.87	35.73	36.00	AC23	0.91	33.27	36.00
AC23	0.93	40.36	37.00	AC7	0.86	41.91	37.00	AC52	0.84	33.82	37.00
AC59	0.92	37.55	38.00	AC24	0.85	29.18	38.00	AC10	0.87	35.64	38.00
AC24	0.92	29.45	39.00	AC47	0.85	39.27	39.00	AC36	0.93	37.91	39.00
AC18	0.92	47.36	40.00	AC10	0.84	31.64	40.00	AC51	0.83	38.27	40.00
AC21	0.91	32.00	41.00	AC40	0.84	44.55	41.00	AC22	0.89	38.45	41.00
AC10	0.90	41.45	42.00	AC48	0.83	46.91	42.00	AC5	0.82	39.27	42.00
AC45	0.89	28.00	43.00	AC59	0.82	41.27	43.00	AC40	0.90	39.45	43.00
AC55	0.89	37.45	44.00	AC22	0.82	45.09	44.00	AC25	0.92	40.00	44.00
AC56	0.88	37.82	45.00	AC45	0.82	32.73	45.00	AC21	0.85	40.27	45.00
AC53	0.87	39.55	46.00	AC16	0.81	46.82	46.00	AC59	0.87	40.91	46.00
AC19	0.87	51.18	47.00	AC52	0.81	34.55	47.00	AC16	0.88	41.55	47.00
AC51	0.87	39.45	48.00	AC57	0.79	41.18	48.00	AC18	0.89	42.09	48.00
AC52	0.87	35.36	49.00	AC51	0.79	38.91	49.00	AC44	0.92	42.27	49.00
AC37	0.86	53.18	50.00	AC21	0.79	45.36	50.00	AC49	0.79	43.45	50.00
AC20	0.86	49.91	51.00	AC49	0.79	35.18	51.00	AC1	0.88	44.82	51.00
AC5	0.85	39.45	52.00	AC44	0.79	51.36	52.00	AC20	0.82	48.55	52.00
AC2	0.85	54.09	53.00	AC20	0.78	48.09	53.00	AC2	0.87	48.64	53.00
AC35	0.84	44.55	54.00	AC5	0.78	40.00	54.00	AC57	0.81	49.36	54.00
AC57	0.82	54.64	55.00	AC19	0.77	54.09	55.00	AC14	0.75	49.91	55.00
AC1	0.82	56.45	56.00	AC14	0.73	45.27	56.00	AC33	0.56	52.82	56.00
AC3	0.79	57.45	57.00	AC3	0.72	54.82	57.00	AC19	0.82	53.27	57.00
AC49	0.79	54.55	58.00	AC37	0.66	57.55	58.00	AC3	0.76	56.55	58.00
AC14	0.77	55.09	59.00	AC35	0.65	57.82	59.00	AC35	0.75	56.73	59.00
AC11	0.75	45.27	60.00	AC33	0.51	56.64	60.00	AC37	0.76	56.82	60.00
AC8	0.64	47.45	61.00	AC30	0.48	60.45	61.00	AC11	0.60	59.82	61.00
AC33	0.62	46.36	62.00	AC11	0.46	61.91	62.00	AC8	0.52	61.09	62.00
AC30	0.53	62.73	63.00	AC8	0.39	62.73	63.00	AC30	0.51	62.73	63.00
AC13	0.37	45.64	64.00	AC13	0.13	64.00	64.00	AC13	0.25	63.73	64.00

**Table 4:** Statistical omics analysis for integrating the responses of tested materials by different biochemical traits in the presence of drought stress.

Treatments	Traits	*p*-Value	Significant	Moderate Tolerance	High Tolerance	Low Tolerance
T1 (7.5% PEG)	PC (µg/g)	0.00	Yes	1158.00 ± 21.22 b	1642.00 ± 42.01a	821.24 ± 27.19 b
	SSC (µg/g)	0.00	Yes	169.04 ±12.13 a	204.13 ± 16.27 b	142.42 ± 17.72 a
	TPC (µg/g)	0.00	Yes	221.24 ± 9.17 a	302.84 ± 25.21 b	151.41 ± 22.61 a
T2 (15% PEG)	PC (µg/g)	0.00	Yes	3281.02 ± 36 a	1880.03 ± 44.67 b	881.18 ± 31.72 c
	SSC (µg/g)	0.00	Yes	276.49 ± 18.28 a	196.86 ± 13.23 b	143.45 ± 14.02 c
	TPC (µg/g)	0.00	Yes	482.73 ± 17.16 a	353.89 ± 22.70 b	241.08 ± 17.06 c

Different letters in a row indicate a significant variation in the mean values, as determined by Duncan’s Multiple-Range Test (*p* ≤ 0.01).

## Data Availability

The article and supplementary files contain all data.

## Supplementary Materials

The following supporting information can be downloaded at: https://www.mdpi.com/article/10.3390/life14111502/s1, Table S1: Means of morphological characters under control (0% PEG) condition; Table S2: Means of morphological characteristics under T1 (7.5% PEG); Table S3: Means of morphological characters under T2 (15% PEG); Table S4: The mean values of morphological traits of all accessions under all drought conditions; Table S5: The interaction values of morphological traits among the tomato accessions and treatments; Table S6: The mean values of biochemical traits under control (T0) condition of drought; Table S7: The mean values of biochemical traits under 7.5% PEG-induced condition; Table S8: The mean values of biochemical traits under 15%PEG-induced condition; Table S9: The mean values of biochemical traits of all accessions under all drought conditions; Table S10: The interaction values of biochemical traits among the tomato accessions and treatments.
