# Supplementary material for: In Vitro Evaluation of Iraqi Kurdistan Tomato Accessions Under Drought Stress Conditions Using Polyethylene Glycol-6000
Source: Life (Basel). 2024 Nov 18;14(11):1502. doi: 10.3390/life14111502 (PMC11595924; doi:10.3390/life14111502)
Supplement: Supplementary file 1 [file life-14-01502-s001.zip › life-3304721-supplementary.pdf]

Table S1. Means of morphological characters under control (0% PEG) condition.

Accessions	GP	RL	SL	FW	DW
AC1	98.33 jkl	3.34 a	4.50 a	38.48 b-h	2.02 g-p
AC2	95.00 h-l	8.30 g-r	6.64 c-k	45.93 d-p	2.47 o-s
AC3	95.00 h-l	6.74 c-f	6.75 d-m	35.77 b-e	1.82 e-k
AC4	100.00 l	8.23 g-r	6.65 c-k	48.53 f-s	1.55 b-g
AC5	85.00 c-f	6.70 cde	7.63 k-s	39.07 b-i	1.85 f-l
AC6	100.00 l	7.57 e-k	7.05 d-p	30.62 b	1.08 abc
AC7	93.33 g-l	8.04 g-q	6.71 d-l	44.50 c-o	1.72 d-h
AC8	73.33 b	5.58 b	7.50 j-s	55.65 n-v	2.57 p-s
AC9	96.67 i-l	7.80 e-n	7.45 i-s	33.65 bc	1.07 ab
AC10	86.67 c-g	8.48 j-s	8.01 p-s	67.07 wx	2.75 rs
AC11	80.00 c	6.36 bcd	6.93 d-n	29.55 a	0.92 a
AC12	88.33 d-h	8.68 k-u	8.19 rs	55.45 n-v	1.90 f-n
AC13	55.00 a	3.12 a	7.37 i-r	56.33 o-w	2.07 g-q
AC14	81.67 cd	7.72 e-m	7.70 m-s	39.65 b-i	1.70 d-h
AC15	96.67 i-l	8.47 j-s	7.09 d-p	61.25 t-x	2.30 j-s
AC16	91.67 e-k	6.02 bc	7.29 g-r	46.78 d-q	2.43 m-s
AC17	95.00 h-l	10.39 yz	8.00 o-s	58.67 r-x	2.09 g-q
AC18	91.67 e-k	8.23 g-r	7.40 i-r	56.27 o-w	2.28 i-r
AC19	88.33 d-h	9.04 n-x	7.16 f-p	64.06 vwx	2.44 n-s
AC20	88.33 d-h	7.22 d-i	6.92 d-n	68.97 x	2.83 s
AC21	88.33 d-h	7.18 d-h	6.85 d-m	52.21 j-u	1.99 g-o
AC22	91.67 e-k	7.39 d-j	6.30 b-f	54.11 l-v	2.29 i-s
AC23	91.67 e-k	8.35 h-r	6.37 b-h	50.76 i-u	1.87 f-m
AC24	88.33 d-h	7.36 d-j	7.24 f-r	54.52 m-v	2.37 k-s
AC25	91.67 e-k	10.00 w-z	7.23 f-q	56.29 o-w	2.41 l-s
AC26	95.00 h-l	9.79 u-y	7.11 e-p	48.30 f-s	1.98 g-o
AC27	91.67 e-k	8.77 h-v	6.34 b-g	33.56 bc	1.80 e-j
AC28	91.67 e-k	9.97 v-z	7.07 d-p	58.93 s-x	2.07 g-q
AC29	91.67 e-k	10.93 z	8.12 qrs	61.92 u-x	2.33 j-s
AC30	71.67 b	9.68 s-y	7.44 i-r	52.33 j-u	2.78 rs
AC31	91.67 e-k	8.96 m-x	6.79 d-m	44.57 c-o	2.06 g-q
AC32	93.33 g-l	9.42 r-y	7.35 h-r	44.54 c-o	1.89 f-n
AC33	69.33 b	8.43 i-r	6.71 d-l	44.16 c-n	2.11 g-q
AC34	94.67 h-l	8.70 k-u	6.82 d-m	45.86 d-p	1.99 g-o
AC35	85.33 c-f	7.69 e-l	6.17 b-e	49.09 g-s	1.99 g-o
AC36	94.67 h-l	8.54 j-t	6.12 bcd	47.79 f-s	1.92 f-o
AC37	93.33 g-l	7.07 c-g	5.68 b	47.87 f-s	1.90 f-n
AC38	92.00 f-k	7.07 c-g	5.76 bc	43.47 c-m	1.68 d-h
AC39	97.33 jkl	8.47 j-s	6.80 d-m	42.93 c-m	1.71 d-h
AC40	92.00 f-k	7.93 f-q	7.04 d-p	47.02 d-q	1.89 f-n
AC41	98.67 kl	8.96 m-x	6.98 d-n	46.01 d-p	1.69 d-h
AC42	96.00 h-l	9.15 p-x	7.70 l-s	41.85 b-k	1.21 a-d
AC43	98.67 kl	8.01 g-q	7.51 j-s	36.73 b-f	1.39 a-f
AC44	94.67 h-l	8.91 l-w	7.35 i-r	61.64 t-x	2.58 qrs
AC45	86.67 c-g	7.18 d-h	6.62 c-j	45.85 d-p	2.06 g-q
AC46	93.33 g-l	8.01 g-q	6.81 d-m	46.52 d-q	2.03 g-q
AC47	90.67 e-j	8.04 g-q	7.55 j-s	35.41 bcd	1.30 a-e
AC48	93.33 g-l	8.16 g-q	7.56 j-s	35.20 bcd	1.19 a-d
AC49	84.00 cde	7.83 e-o	6.78 d-m	50.12 h-t	1.92 f-o
AC50	90.67 e-j	9.18 q-x	8.12 qrs	37.19 b-g	1.55 b-g
AC51	84.00 cde	8.60 j-u	7.69 l-s	58.11 q-x	2.29 i-s
AC52	85.33 c-f	7.91 e-p	6.64 c-k	39.07 b-i	1.85 f-l
AC53	85.33 c-f	8.59 j-u	6.90 d-n	47.33 e-s	1.73 d-i
AC54	89.33 e-i	9.01 n-x	7.57 j-s	41.39 b-j	1.69 d-h
AC55	86.67 c-g	8.13 g-q	6.74 d-m	47.27 e-r	1.79 e-j
AC56	85.33 c-f	9.07 o-x	6.49 b-i	41.18 b-j	1.79 e-j
AC57	84.00 cde	9.73 t-y	7.70 m-s	39.62 b-i	1.97 g-o
AC58	93.33 g-l	8.72 k-u	7.50 j-s	56.46 p-w	2.31 j-s
AC59	89.33 e-i	7.93 f-q	7.26 f-r	46.35 d-p	1.99 g-o
AC60	96.00 h-l	10.08 w-z	7.02 d-o	53.40 k-v	2.05 g-q
AC61	97.33 jkl	8.58 j-u	7.11 e-p	39.14 b-i	1.59 c-h
AC62	92.00 f-k	8.45 i-s	6.87 d-m	42.64 c-l	2.35 j-s
AC63	97.33 jkl	9.04 n-x	8.41 s	41.11 b-j	1.63 d-h
AC64	97.33 jkl	10.16 xyz	7.86 n-s	44.05 c-n	2.15 h-q

GP: germination percentage, RL: root length, SL: shoot length, FW: fresh weight, DW: Dry weight. Distinct letters indicate a statistically significant difference between the mean values as per Duncan's Multiple-Range Test ($p \leq 0.01$).

Table S2. Means of morphological characteristics under T1 (7.5% PEG).

Accessions	RL	SL	FW	DW	GP
AC1	2.92 v	4.27 o-r	41.30 d-l	3.52 a-d	78.33 h-k
AC2	2.70 v	4.57 m-r	41.32 d-l	2.65 f-p	78.33 h-k
AC3	5.46 n-t	5.82 c-j	35.78 j-r	2.40 h-s	70.00 k
AC4	6.47 c-o	4.86 i-r	42.90 c-j	2.37 h-s	100.00 a
AC5	5.69 j-t	6.39 a-g	33.00 m-s	1.83 r-u	83.33 e-i
AC6	5.52 m-t	5.38 f-n	26.25 st	1.90 q-u	100.00 a
AC7	6.20 f-p	6.16 a-h	37.12 h-r	2.32 j-s	91.67 a-f
AC8	4.56 tu	4.52 m-r	45.45 b-g	3.57 abc	73.33 jk
AC9	6.99 a-i	6.15 a-h	39.35 f-p	2.80 d-o	96.67 abc
AC10	6.69 b-n	6.58 a-d	56.78 a	3.92 a	83.33 e-i
AC11	5.49 n-t	5.50 e-m	30.68 q-t	1.43 tu	78.33 h-k
AC12	7.49 a-d	6.34 a-g	39.88 e-n	3.15 b-g	85.00 d-i
AC13	2.21 v	5.84 c-i	44.15 c-h	2.95 c-k	55.00 l
AC14	4.83 r-u	4.86 i-r	32.20 m-t	2.27 k-s	78.33 h-k
AC15	7.44 a-e	4.60 l-r	48.23 bcd	3.72 ab	85.00 d-i
AC16	4.98 q-u	6.03 c-h	37.02 h-r	2.85 c-n	88.33 b-h
AC17	7.57 abc	5.09 h-q	46.90 b-f	2.93 c-l	88.33 b-h
AC18	5.80 i-s	3.98 r	25.31 t	2.15 m-s	85.00 d-i
AC19	6.99 a-i	5.35 g-o	44.20 c-h	3.09 b-i	80.00 g-j
AC20	6.64 b-n	4.64 k-r	52.19 ab	3.75 ab	81.67 f-j
AC21	6.16 f-q	5.68 d-l	48.31 bcd	3.19 b-g	86.67 c-i
AC22	5.53 m-t	4.31 n-r	47.68 b-e	2.79 d-o	90.00 a-g
AC23	5.63 k-t	5.12 h-p	36.27 h-r	2.25 k-s	86.67 c-i
AC24	5.69 j-t	5.12 h-p	42.92 c-j	2.41 h-s	88.33 b-h
AC25	6.55 c-n	5.14 h-p	44.07 c-i	2.85 c-n	85.00 d-i
AC26	6.92 a-j	6.08 b-h	38.55 g-q	2.35 i-s	93.33 a-e
AC27	6.95 a-i	5.55 d-m	31.67 o-t	2.30 j-s	91.67 a-f
AC28	6.66 b-n	5.38 f-n	45.90 b-g	3.20 b-g	90.00 a-g
AC29	6.92 a-j	5.14 h-p	42.05 c-k	3.49 a-e	90.00 a-g
AC30	4.23 u	4.12 pqr	44.14 c-h	3.25 a-f	58.33 l
AC31	6.38 c-p	5.07 h-q	39.75 e-o	2.73 f-p	91.67 a-f
AC32	6.92 a-j	6.46 a-f	39.93 e-n	2.47 g-r	90.00 a-g
AC33	4.18 u	4.75 j-r	35.63 j-r	2.49 g-r	72.00 jk
AC34	6.81 a-l	5.92 c-i	39.51 f-p	1.99 p-u	89.33 b-g
AC35	5.18 p-u	4.88 i-r	42.75 c-j	2.92 c-l	84.00 e-i
AC36	6.48 c-o	5.13 h-p	43.22 c-j	2.77 e-o	86.67 c-i
AC37	5.31 o-u	4.55 m-r	42.43 c-j	2.76 e-o	80.00 g-j
AC38	5.89 h-s	4.89 i-r	38.83 f-p	2.39 h-s	92.00 a-f
AC39	5.95 h-r	4.53 m-r	37.23 h-r	2.47 g-r	96.00 abc
AC40	6.73 b-m	5.34 g-o	41.99 c-k	2.73 f-p	86.67 c-i
AC41	6.38 c-p	5.60 d-m	39.55 f-o	2.05 o-t	92.00 a-f
AC42	6.75 b-m	6.15 a-h	35.95 i-r	1.35 u	96.00 abc
AC43	6.90 a-j	7.15 a	33.29 l-s	2.15 m-s	94.67 a-d
AC44	6.94 a-i	5.98 c-h	49.81 bc	2.99 c-k	92.00 a-f
AC45	6.34 c-p	5.52 d-m	41.69 d-k	2.63 f-p	86.67 c-i
AC46	6.05 g-q	5.43 f-m	38.44 g-q	2.19 l-s	93.33 a-e
AC47	7.39 a-f	7.10 ab	29.71 rst	2.13 n-s	94.67 a-d
AC48	7.49 a-d	7.11 ab	31.93 n-t	1.83 r-u	92.00 a-f
AC49	6.44 c-o	5.57 d-m	41.29 d-l	2.25 k-s	77.33 ijk
AC50	7.84 ab	6.56 a-e	33.34 l-s	2.17 m-s	86.67 c-i
AC51	6.22 e-p	5.71 d-k	36.57 h-r	2.17 m-s	84.00 e-i
AC52	6.55 c-n	5.79 c-j	40.08 e-m	2.48 g-r	84.00 e-i
AC53	6.80 a-l	5.31 g-o	36.93 h-r	3.09 b-h	84.00 e-i
AC54	7.98 a	7.20 a	32.03 m-t	2.31 j-s	88.00 b-h
AC55	6.32 d-p	5.15 h-p	38.61 g-q	2.98 c-k	85.33 d-i
AC56	6.60 c-n	5.87 c-i	38.13 g-q	2.77 e-o	84.00 e-i
AC57	5.52 m-t	5.56 d-m	35.95 i-r	2.89 c-m	78.67 h-k
AC58	4.70 stu	4.04 qr	37.97 g-q	2.65 f-p	89.33 b-g
AC59	5.94 h-r	5.55 d-m	37.10 h-r	3.05 b-j	86.67 c-i
AC60	6.85 a-k	5.80 c-j	42.33 c-j	2.32 j-s	93.33 a-e
AC61	7.20 a-g	6.80 abc	31.40 p-t	1.67 stu	97.33 ab
AC62	7.25 a-g	6.78 abc	36.19 h-r	2.54 f-r	89.33 b-g
AC63	7.10 a-h	6.81 abc	34.15 k-r	2.29 k-s	93.33 a-e
AC64	5.58 l-t	4.58 m-r	35.87 j-r	2.49 g-r	94.67 a-d

GP: germination percentage, RL: root length, SL: shoot length, FW: fresh weight, DW: dry weight. Different letters signify a statistically significant difference between the mean values according to Duncan's Multiple-Range Test ($p \leq 0.01$).

Table S3. Means of morphological characters under T2 (15% PEG).

Accessions	RL		SL		FW		DW		GP	
AC1	3.58	t-z	2.70	stu	28.87	i-v	4.11	abc	91.67	abc
AC2	4.29	m-w	3.46	k-t	23.82	u-x	2.75	k-x	83.33	a-h
AC3	2.74	y-aa	2.96	q-u	28.02	j-x	3.53	c-k	68.33	i-l
AC4	2.82	y-aa	1.83	vw	32.68	a-p	2.93	g-u	90.00	a-e
AC5	3.09	xyz	2.43	uv	16.72	z	2.43	o-z	81.67	b-h
AC6	3.11	xyz	3.05	o-u	26.58	p-x	3.35	c-m	91.67	abc
AC7	4.41	l-v	3.80	f-q	28.90	i-v	2.80	i-w	80.00	c-i
AC8	2.01	aa	1.36	wx	34.12	a-k	5.00	a	46.67	n
AC9	5.63	d-k	4.07	c-m	34.18	a-j	3.48	c-k	90.00	a-e
AC10	3.86	q-y	3.61	h-s	33.22	a-o	4.42	ab	83.33	a-h
AC11	3.85	r-y	3.81	f-q	24.52	t-x	1.80	yz	46.67	n
AC12	6.01	d-h	4.45	b-i	28.93	i-v	3.03	f-s	85.00	a-g
AC13	0.83	ab	1.00	x	17.55	yz	3.10	f-r	20.00	o
AC14	3.68	s-z	3.43	k-t	25.28	s-x	2.93	g-u	76.67	f-j
AC15	5.96	d-h	3.05	o-u	36.40	a-f	3.62	c-i	88.33	a-f
AC16	3.65	t-z	4.16	c-l	28.37	j-w	2.20	t-z	78.33	d-j
AC17	5.93	d-i	2.95	q-u	33.73	a-m	3.31	c-m	81.67	a-h
AC18	5.17	g-o	3.43	k-t	37.97	abc	3.83	b-f	81.67	a-h
AC19	6.32	c-f	3.28	l-u	37.01	a-d	3.99	b-e	71.67	h-k
AC20	6.03	d-h	3.88	e-q	38.32	ab	4.42	ab	75.00	g-j
AC21	4.71	j-t	2.62	tuv	32.58	a-p	3.06	f-s	78.33	d-j
AC22	5.15	g-p	3.28	l-u	28.59	j-v	2.99	g-t	78.33	d-j
AC23	4.64	j-t	3.42	k-t	27.79	m-x	2.54	m-y	85.00	a-g
AC24	5.23	f-n	3.49	i-t	27.89	l-x	2.99	g-t	83.33	a-h
AC25	5.06	g-p	3.92	d-p	38.31	ab	3.37	c-l	81.67	a-h
AC26	3.21	w-z	4.00	c-o	23.71	u-x	2.48	n-y	90.00	a-e
AC27	3.20	w-z	3.49	i-t	22.44	wxy	2.57	l-y	91.67	abc
AC28	5.22	f-n	3.47	j-t	36.79	a-e	3.60	c-j	86.67	a-g
AC29	5.09	g-p	3.89	e-q	34.08	a-l	3.37	c-l	85.00	a-g
AC30	3.72	s-z	2.94	q-u	30.70	e-s	4.01	a-d	53.33	mn
AC31	4.99	g-q	3.29	l-u	30.85	e-s	3.19	e-o	88.33	a-f
AC32	4.81	i-s	3.60	h-s	29.21	h-v	3.14	f-p	86.67	a-g
AC33	3.32	v-z	3.07	n-u	33.83	a-m	3.00	g-t	60.00	lm
AC34	4.92	g-r	3.60	h-s	28.65	j-v	2.33	p-z	85.33	a-g
AC35	3.47	u-z	3.11	m-u	30.30	f-t	2.78	j-x	66.67	j-l
AC36	4.91	h-r	3.87	e-q	35.23	a-h	3.69	b-g	81.33	b-h
AC37	3.64	t-z	2.76	r-u	31.23	d-s	3.69	b-g	62.67	klm
AC38	4.08	o-x	3.07	n-u	26.15	q-x	2.79	j-x	90.67	a-d
AC39	5.15	g-p	2.93	q-u	26.87	o-x	2.81	h-w	90.67	a-d
AC40	4.68	j-t	3.78	f-q	36.37	a-f	3.36	c-m	78.67	c-i
AC41	4.02	p-x	3.53	i-t	27.96	k-x	2.32	q-z	86.67	a-g
AC42	4.11	n-x	4.07	c-m	22.09	xy	1.69	z	85.33	a-g
AC43	5.44	e-l	4.26	b-k	23.37	vwx	2.26	s-z	86.67	a-g
AC44	3.82	r-y	3.17	m-u	35.55	a-g	3.32	c-m	72.00	h-k
AC45	5.17	g-o	5.15	b	33.05	a-p	3.13	f-q	80.00	b-i
AC46	4.11	n-x	4.01	c-n	32.54	a-q	2.84	h-v	84.00	a-h
AC47	4.19	n-x	4.31	b-k	26.99	o-x	2.03	w-z	80.00	b-i
AC48	4.53	k-u	4.31	b-k	25.47	r-x	1.99	xyz	76.00	f-j
AC49	4.07	o-x	3.52	i-t	31.96	c-q	3.09	f-r	81.33	b-h
AC50	7.24	abc	4.23	c-l	29.10	h-v	2.91	g-u	86.67	a-g
AC51	2.69	zaa	3.87	e-q	33.43	a-n	3.39	c-k	81.33	b-h
AC52	4.65	j-t	3.69	g-r	31.53	d-r	3.27	d-n	81.33	b-h
AC53	6.37	cde	4.92	bc	32.23	b-q	3.63	c-h	85.33	a-g
AC54	7.45	ab	4.70	b-f	29.52	g-u	3.33	c-m	90.67	a-d
AC55	7.22	abc	4.62	b-g	32.05	c-q	2.29	r-z	86.67	a-g
AC56	8.02	a	4.77	b-e	34.87	a-i	2.04	v-z	84.00	a-h
AC57	5.10	g-p	4.50	b-h	30.79	e-s	3.39	c-k	77.33	e-j
AC58	3.18	w-z	3.47	j-t	32.24	b-q	3.07	f-s	90.67	a-d
AC59	5.74	d-j	4.44	b-i	27.26	n-x	3.07	f-s	77.33	e-j
AC60	5.33	e-m	4.86	bcd	38.39	a	3.85	b-f	78.67	c-i
AC61	6.34	cde	4.42	b-j	26.13	q-x	2.15	u-z	94.67	a
AC62	6.65	bcd	4.43	b-i	29.45	g-v	3.59	c-j	80.00	b-i
AC63	6.06	d-g	6.07	a	26.72	p-x	2.29	r-z	92.00	ab
AC64	4.02	p-x	3.00	p-u	36.33	a-f	3.15	f-o	88.00	a-g

GP: germination percentage, RL: root length, SL: shoot length, FW: fresh weight, DW: dry weight. Different letters denote a statistically significant variation between the mean values determined by Duncan's Multiple-Range Test ($p \leq 0.01$).

Table S4. The mean values of morphological traits of all accessions under all drought conditions.

Accessions	GP		RL		SL		FW		DW	
AC1	89.44	e-m	3.28	a1	3.82	b1	36.22	v-b1	3.22	cd
AC2	85.56	l-s	5.10	wxy	4.89	t-z	37.02	t-a1	2.62	h-n
AC3	77.78	w	4.98	xy	5.17	m-v	33.19	a1-d1	2.58	h-q
AC4	96.67	a	5.84	q-u	4.44	za1	41.37	j-s	2.28	o-w
AC5	83.33	p-v	5.16	v-y	5.48	f-o	29.59	d1-f1	2.04	v-y
AC6	97.22	a	5.40	t-y	5.16	m-v	27.82	f1	2.11	u-y
AC7	88.33	g-o	6.22	l-r	5.56	e-m	36.84	u-a1	2.28	p-w
AC8	64.44	xy	4.05	z	4.46	y-a1	45.07	c-j	3.71	a
AC9	94.44	a-d	6.81	f-j	5.89	c-g	35.73	w-b1	2.45	k-t
AC10	84.44	n-t	6.34	j-q	6.07	bcd	52.36	ab	3.69	a
AC11	68.33	x	5.23	v-y	5.41	h-q	28.25	e1f1	1.38	a1
AC12	86.11	k-r	7.39	cde	6.33	bc	41.42	j-s	2.69	g-l
AC13	43.33	z	2.05	b1	4.74	v-a1	39.34	n-w	2.71	g-l
AC14	78.89	uvw	5.41	t-y	5.33	j-t	32.38	b1-d1	2.30	n-w
AC15	90.00	c-l	7.29	def	4.91	s-y	48.63	bcd	3.21	cd
AC16	86.11	k-r	4.88	y	5.82	d-h	37.39	s-z	2.49	j-t
AC17	88.33	g-o	7.97	ab	5.35	i-t	46.44	c-g	2.78	f-j
AC18	86.11	k-r	6.40	j-p	4.94	r-x	39.85	m-v	2.76	f-k
AC19	80.00	t-w	7.45	b-e	5.26	l-u	48.42	b-e	3.17	cde
AC20	81.67	r-w	6.63	i-m	5.15	m-v	53.16	a	3.67	ab
AC21	84.44	n-t	6.01	o-s	5.05	n-w	44.37	e-l	2.75	f-k
AC22	86.67	j-q	6.02	o-s	4.63	w-a1	43.46	f-m	2.69	g-l
AC23	87.78	h-q	6.21	l-r	4.97	q-x	38.27	p-x	2.22	r-x
AC24	86.67	j-q	6.09	m-r	5.28	k-u	41.78	j-r	2.59	h-q
AC25	86.11	k-r	7.20	d-h	5.43	h-p	46.22	c-h	2.87	e-h
AC26	92.78	a-g	6.64	i-m	5.73	d-k	36.85	u-a1	2.27	q-w
AC27	91.67	b-i	6.31	j-q	5.13	m-v	29.22	d1-f1	2.22	r-x
AC28	89.44	e-m	7.28	def	5.31	j-t	47.21	c-f	2.96	d-g
AC29	88.89	f-n	7.65	a-d	5.72	d-l	46.02	c-i	3.06	c-f
AC30	61.11	y	5.88	p-t	4.83	u-z	42.39	g-o	3.35	bc
AC31	90.56	c-k	6.78	f-k	5.05	n-w	38.39	o-x	2.66	g-m
AC32	90.00	c-l	7.05	e-i	5.80	d-i	37.89	r-y	2.50	j-t
AC33	67.11	x	5.31	u-y	4.84	u-z	37.87	r-y	2.53	i-s
AC34	89.78	d-l	6.81	f-j	5.45	g-p	38.01	q-y	2.11	u-y
AC35	78.67	vw	5.45	t-x	4.72	v-a1	40.71	k-u	2.56	h-q
AC36	87.56	i-q	6.64	i-m	5.04	o-w	42.08	i-q	2.79	f-j
AC37	78.67	vw	5.34	t-y	4.33	a1	40.51	l-u	2.78	f-j
AC38	91.56	c-i	5.68	r-v	4.57	x-a1	36.15	v-b1	2.28	o-w
AC39	94.67	abc	6.53	i-o	4.75	v-a1	35.68	w-b1	2.33	n-w
AC40	85.78	k-r	6.45	j-o	5.39	h-r	41.80	j-r	2.66	g-m
AC41	92.44	a-h	6.45	j-o	5.37	h-s	37.84	r-y	2.02	wxy
AC42	92.44	a-h	6.67	h-l	5.97	cde	33.29	z-d1	1.42	a1
AC43	93.33	a-f	6.78	f-k	6.31	bc	31.13	c1-f1	1.94	xyz
AC44	86.22	k-r	6.56	i-o	5.50	f-n	49.00	bc	2.96	d-g
AC45	84.44	n-t	6.23	k-r	5.76	d-j	40.19	m-v	2.61	h-o
AC46	90.22	c-l	6.05	n-s	5.42	h-q	39.17	n-w	2.36	m-v
AC47	88.44	g-n	6.54	i-o	6.32	bc	30.70	c1-f1	1.82	yz
AC48	87.11	i-q	6.73	g-l	6.32	bc	30.87	c1-f1	1.67	za1
AC49	80.89	sw	6.11	m-r	5.29	k-u	41.12	j-t	2.42	l-u
AC50	88.00	g-o	8.09	a	6.30	bc	33.21	a1-d1	2.21	s-x
AC51	83.11	q-v	5.83	q-u	5.75	d-j	42.70	g-n	2.62	h-n
AC52	83.56	o-u	6.37	j-q	5.37	h-s	36.90	u-a1	2.54	i-r
AC53	84.89	m-s	7.25	d-g	5.71	d-l	38.83	n-w	2.82	f-j
AC54	89.33	f-m	8.14	a	6.49	b	34.31	x-c1	2.44	k-t
AC55	86.22	k-r	7.23	d-g	5.50	f-n	39.31	n-w	2.35	m-v
AC56	84.44	n-t	7.90	abc	5.71	d-l	38.06	q-y	2.20	t-x
AC57	80.00	t-w	6.79	f-j	5.92	c-f	35.45	w-b1	2.75	f-k
AC58	91.11	c-j	5.54	s-w	5.01	p-x	42.22	h-p	2.68	g-m
AC59	84.44	n-t	6.54	i-o	5.75	d-j	36.90	u-a1	2.70	g-l
AC60	89.33	f-m	7.42	b-e	5.89	c-g	44.71	d-k	2.74	f-l
AC61	96.44	ab	7.37	cde	6.11	bcd	32.22	b1-e1	1.81	yz
AC62	87.11	i-q	7.45	b-e	6.03	cd	36.09	v-b1	2.83	f-i
AC63	94.22	a-e	7.40	cde	7.10	a	33.99	y-c1	2.07	v-y
AC64	93.33	a-f	6.58	i-n	5.15	m-v	38.75	n-w	2.60	h-p

GP: germination percentage, RL: root length, SL: shoot length, FW: fresh weight, DW: dry weight. Different letters indicate a statistically significant difference between mean values assessed by Duncan's Multiple-Range Test ($p < 0.01$).

Table S5. The interaction values of morphological traits among the tomato accessions and treatments.

Acc. X treat.	GP		RL		SL		FW		DW	
AC1,Control	98.33	ab	3.34	a3-j3	4.50	b2-l2	38.48	c1-a2	2.02	u1-m2
AC2,Control	95.00	a-d	8.30	i-u	6.64	m-c1	45.93	m-b1	2.47	f1-x1
AC3,Control	95.00	a-d	6.74	d1-p1	6.75	j-a1	35.77	j1-i2	1.82	c2-q2
AC4,Control	100.00	a	8.23	i-u	6.65	l-c1	48.53	i-r	1.55	m2-t2
AC5,Control	85.00	g-l	6.70	e1-p1	7.63	a-h	39.07	a1-x1	1.85	a2-q2
AC6,Control	100.00	a	7.57	q-e1	7.05	f-u	30.62	c2-w2	1.08	s2-u2
AC7,Control	93.33	a-f	8.04	k-x	6.71	k-b1	44.50	n-d1	1.72	f2-r2
AC8,Control	73.33	o-t	5.58	u1-i2	7.50	b-j	55.65	d-h	2.57	c1-u1
AC9,Control	96.67	abc	7.80	n-b1	7.45	b-k	33.65	r1-p2	1.07	t2u2
AC10,Control	86.67	e-k	8.48	h-q	8.01	a-d	67.07	ab	2.75	x-q1
AC11,Control	80.00	j-p	6.36	h1-x1	6.93	f-v	29.55	e2-y2	0.92	u2
AC12,Control	88.33	d-i	8.68	h-o	8.19	ab	55.45	d-i	1.90	y1-p2
AC13,Control	55.00	wx	3.12	d3-k3	7.37	c-n	56.33	d-g	2.07	t1-m2
AC14,Control	81.67	i-n	7.72	o-c1	7.70	a-f	39.65	y-t1	1.70	h2-r2
AC15,Control	96.67	abc	8.47	h-q	7.09	e-u	61.25	b-e	2.30	k1-d2
AC16,Control	91.67	b-g	6.02	k1-c2	7.29	d-o	46.78	l-x	2.43	g1-z1
AC17,Control	95.00	a-d	10.39	Ab	7.92	a-d	58.67	c-f	2.09	s1-m2
AC18,Control	91.67	b-g	8.23	i-u	7.40	b-m	56.27	d-g	2.28	l1-f2
AC19,Control	88.33	d-i	9.04	e-j	7.16	e-q	64.06	abc	2.44	f1-y1
AC20,Control	88.33	d-i	7.22	v-h1	6.92	f-v	68.97	a	2.83	s-m1
AC21,Control	88.33	d-i	7.18	w-h1	6.85	h-y	52.21	f-m	1.99	v1-n2
AC22,Control	91.67	b-g	7.39	u-g1	6.30	u-k1	54.11	f-k	2.29	k1-e2
AC23,Control	91.67	b-g	8.35	i-t	6.37	s-i1	50.76	g-n	1.87	z1-p2
AC24,Control	88.33	d-i	7.36	u-g1	7.24	d-q	54.52	e-j	2.37	i1-c2
AC25,Control	91.67	b-g	10.00	a-e	7.23	d-q	56.29	d-g	2.41	h1-a1
AC26,Control	95.00	a-d	9.79	b-f	7.11	e-t	48.30	j-s	1.98	v1-n2
AC27,Control	91.67	b-g	8.77	g-m	6.34	t-j1	33.56	r1-p2	1.80	d2-q2
AC28,Control	91.67	b-g	9.97	b-e	7.07	f-u	58.93	c-f	2.07	t1-m2
AC29,Control	91.67	b-g	10.93	a	8.12	abc	61.92	a-d	2.33	j1-d2
AC30,Control	71.67	q-t	9.68	b-g	7.44	b-l	52.33	f-m	2.78	v-o1
AC31,Control	91.67	b-g	8.96	f-k	6.79	i-z	44.57	n-d1	2.06	u1-m2
AC32,Control	93.33	a-f	9.42	c-h	7.35	c-o	44.54	n-d1	1.89	y1-p2
AC33,Control	69.33	r-u	8.43	i-s	6.71	k-b1	44.16	n-e1	2.11	s1-m2
AC34,Control	94.67	a-e	8.70	h-n	6.82	i-z	45.86	m-b1	1.99	v1-n2
AC35,Control	85.33	f-l	7.69	p-d1	6.17	v-l1	49.09	h-q	1.99	v1-n2
AC36,Control	94.67	a-e	8.54	h-p	6.12	w-m1	47.79	j-u	1.92	x1-o2
AC37,Control	93.33	a-f	7.07	y-i1	5.68	h1-r1	47.87	j-u	1.90	y1-p2
AC38,Control	92.00	a-g	7.07	y-i1	5.76	g1-p1	43.47	o-g1	1.68	k2-r2
AC39,Control	97.33	abc	8.47	h-q	6.80	i-z	42.93	p-i1	1.71	g2-r2
AC40,Control	92.00	a-g	7.93	m-z	7.04	f-u	47.02	k-v	1.89	y1-p2
AC41,Control	98.67	ab	8.96	f-k	6.98	f-u	46.01	m-a1	1.69	i2-r2
AC42,Control	96.00	a-d	9.15	d-i	7.70	a-f	41.85	r-o1	1.21	r2-u2
AC43,Control	98.67	ab	8.01	k-y	7.51	b-j	36.73	g1-d2	1.39	o2-u2
AC44,Control	94.67	a-e	8.91	f-l	7.35	c-o	61.64	bcd	2.58	b1-u1
AC45,Control	86.67	e-k	7.18	w-h1	6.62	m-d1	45.85	m-b1	2.06	u1-m2
AC46,Control	93.33	a-f	8.01	k-y	6.81	i-z	46.52	l-y	2.03	u1-m2
AC47,Control	90.67	b-h	8.04	k-x	7.55	b-i	35.41	l1-j2	1.30	q2-u2
AC48,Control	93.33	a-f	8.16	j-v	7.56	b-i	35.20	n1-k2	1.19	r2-u2
AC49,Control	84.00	g-m	7.83	m-a1	6.78	i-z	50.12	g-o	1.92	x1-o2
AC50,Control	90.67	b-h	9.18	d-i	8.14	abc	37.19	e1-c2	1.55	m2-t2
AC51,Control	84.00	g-m	8.60	h-p	7.69	a-g	58.11	c-f	2.29	k1-e2
AC52,Control	85.33	f-l	7.91	m-z	6.64	m-d1	39.07	a1-w1	1.85	a2-q2
AC53,Control	85.33	f-l	8.59	h-p	6.90	g-w	47.33	k-u	1.73	e2-r2
AC54,Control	89.33	c-i	9.01	f-j	7.57	b-i	41.39	s-o1	1.69	i2-r2
AC55,Control	86.67	e-k	8.13	j-w	6.74	j-a1	47.27	k-u	1.79	d2-q2
AC56,Control	85.33	f-l	9.07	e-j	6.49	p-g1	41.18	t-q1	1.79	d2-q2
AC57,Control	84.00	g-m	9.73	b-f	7.70	a-f	39.62	y-t1	1.97	w1-n2
AC58,Control	93.33	a-f	8.72	h-n	7.50	b-j	56.46	d-g	2.31	k1-d2
AC59,Control	89.33	c-i	7.93	m-z	7.26	d-p	46.35	l-z	1.99	v1-n2
AC60,Control	96.00	a-d	10.08	a-d	7.02	f-u	53.40	f-l	2.05	u1-m2
AC61,Control	97.33	abc	8.58	h-p	7.11	e-t	39.14	a1-v1	1.59	l2-t2
AC62,Control	92.00	a-g	8.45	i-r	6.87	h-x	42.64	q-k1	2.35	j1-d2
AC63,Control	97.33	abc	9.04	e-j	8.41	a	41.11	u-q1	1.63	k2-s2
AC64,Control	97.33	abc	10.16	abc	7.86	a-e	44.05	n-f1	2.15	s1-l2
AC1,PEG-7.5	78.33	l-q	2.92	g3-l3	4.27	d2-p2	41.30	s-o1	3.52	d-o
AC2,PEG-7.5	78.33	l-q	2.70	j3-l3	4.57	y1-l2	41.32	s-o1	2.65	y-s1

AC3,PEG-7.5	70.00	r-u	5.46	x1-k2	5.82	e1-p1	35.78	j1-i2	2.40	h1-a2
AC4,PEG-7.5	100.00	a	6.47	g1-v1	4.86	t1-f2	42.90	p-i1	2.37	i1-c2
AC5,PEG-7.5	83.33	h-m	5.69	r1-g2	6.39	r-i1	33.00	r1-r2	1.83	b2-q2
AC6,PEG-7.5	100.00	a	5.52	v1-j2	5.38	l1-x1	26.25	q2-z2	1.90	y1-p2
AC7,PEG-7.5	91.67	b-g	6.20	i1-y1	6.16	v-l1	37.12	e1-c2	2.32	k1-d2
AC8,PEG-7.5	73.33	o-t	4.56	k2-x2	4.52	a2-l2	45.45	m-c1	3.57	c-n
AC9,PEG-7.5	96.67	abc	6.99	z-j1	6.15	v-l1	39.35	z-v1	2.80	t-o1
AC10,PEG-7.5	83.33	h-m	6.69	e1-q1	6.58	n-e1	56.78	d-g	3.92	b-e
AC11,PEG-7.5	78.33	l-q	5.49	w1-k2	5.50	l1-v1	30.68	c2-w2	1.43	n2-u2
AC12,PEG-7.5	85.00	g-l	7.49	s-f1	6.34	t-j1	39.88	w-r1	3.15	j-a1
AC13,PEG-7.5	55.00	wx	2.21	l3	5.84	d1-p1	44.15	n-e1	2.95	p-h1
AC14,PEG-7.5	78.33	l-q	4.83	g2-t2	4.86	t1-f2	32.20	v1-t2	2.27	m1-g2
AC15,PEG-7.5	85.00	g-l	7.44	t-f1	4.60	x1-j2	48.23	j-t	3.72	c-i
AC16,PEG-7.5	88.33	d-i	4.98	e2-s2	6.03	z-n1	37.02	f1-c2	2.85	r-k1
AC17,PEG-7.5	88.33	d-i	7.57	q-e1	5.09	p1-c2	46.90	l-w	2.93	p-h1
AC18,PEG-7.5	85.00	g-l	5.80	p1-f2	3.98	h2-t2	25.31	t2-z2	2.15	s1-l2
AC19,PEG-7.5	80.00	j-p	6.99	z-j1	5.35	m1-y1	44.20	n-e1	3.09	k-c1
AC20,PEG-7.5	81.67	i-n	6.64	e1-s1	4.64	w1-j2	52.19	f-m	3.75	c-h
AC21,PEG-7.5	86.67	e-k	6.16	i1-z1	5.68	h1-s1	48.31	j-s	3.19	h-z
AC22,PEG-7.5	90.00	c-h	5.53	u1-i2	4.31	c2-n2	47.68	j-u	2.79	u-o1
AC23,PEG-7.5	86.67	e-k	5.63	t1-h2	5.12	p1-b2	36.27	h1-g2	2.25	o1-i2
AC24,PEG-7.5	88.33	d-i	5.69	s1-g2	5.12	p1-b2	42.92	p-i1	2.41	h1-a2
AC25,PEG-7.5	85.00	g-l	6.55	f1-t1	5.14	o1-b2	44.07	n-f1	2.85	r-k1
AC26,PEG-7.5	93.33	a-f	6.92	a1-l1	6.08	x-n1	38.55	c1-z1	2.35	j1-d2
AC27,PEG-7.5	91.67	b-g	6.95	a1-k1	5.55	j1-u1	31.67	z1-t2	2.30	k1-d2
AC28,PEG-7.5	90.00	c-h	6.66	e1-q1	5.38	l1-x1	45.90	m-b1	3.20	h-z
AC29,PEG-7.5	90.00	c-h	6.92	a1-k1	5.14	o1-b2	42.05	q-n1	3.49	d-p
AC30,PEG-7.5	58.33	wx	4.23	o2-a3	4.12	e2-s2	44.14	n-e1	3.25	h-y
AC31,PEG-7.5	91.67	b-g	6.38	h1-x1	5.07	p1-c2	39.75	x-s1	2.73	y-q1
AC32,PEG-7.5	90.00	c-h	6.92	a1-k1	6.46	g-h1	39.93	v-r1	2.47	f1-x1
AC33,PEG-7.5	72.00	p-t	4.18	q2-b3	4.75	v1-h2	35.63	k1-j2	2.49	d1-w1
AC34,PEG-7.5	89.33	c-i	6.81	c1-o1	5.92	b1-o1	39.51	y-t1	1.99	v1-n2
AC35,PEG-7.5	84.00	g-m	5.18	a2-n2	4.88	s1-e2	42.75	p-j1	2.92	q-i1
AC36,PEG-7.5	86.67	e-k	6.48	g1-u1	5.13	o1-b2	43.22	o-h1	2.77	w-o1
AC37,PEG-7.5	80.00	j-p	5.31	y1-m2	4.55	z1-l2	42.43	q-l1	2.76	w-p1
AC38,PEG-7.5	92.00	a-g	5.89	o1-e2	4.89	r1-e2	38.83	b1-y1	2.39	h1-b2
AC39,PEG-7.5	96.00	a-d	5.95	m1-c2	4.53	a2-l2	37.23	e1-c2	2.47	f1-x1
AC40,PEG-7.5	86.67	e-k	6.73	e1-p1	5.34	m1-z1	41.99	q-n1	2.73	y-r1
AC41,PEG-7.5	92.00	a-g	6.38	h1-x1	5.60	i1-t1	39.55	y-t1	2.05	u1-m2
AC42,PEG-7.5	96.00	a-d	6.75	d1-p1	6.15	v-l1	35.95	i1-i2	1.35	p2-u2
AC43,PEG-7.5	94.67	a-e	6.90	a1-m1	7.15	e-s	33.29	r1-q2	2.15	s1-l2
AC44,PEG-7.5	92.00	a-g	6.94	a1-k1	5.98	a1-n1	49.81	g-p	2.99	o-g1
AC45,PEG-7.5	86.67	e-k	6.34	h1-x1	5.52	k1-v1	41.69	r-o1	2.63	a1-t1
AC46,PEG-7.5	93.33	a-f	6.05	j1-b2	5.43	l1-w1	38.44	c1-a2	2.19	q1-k2
AC47,PEG-7.5	94.67	a-e	7.39	u-g1	7.10	e-t	29.71	d2-x2	2.13	s1-l2
AC48,PEG-7.5	92.00	a-g	7.49	r-f1	7.11	e-t	31.93	y1-t2	1.83	b2-q2
AC49,PEG-7.5	77.33	l-r	6.44	g1-w1	5.57	j1-u1	41.29	s-p1	2.25	o1-i2
AC50,PEG-7.5	86.67	e-k	7.84	m-a1	6.56	o-f1	33.34	r1-q2	2.17	r1-k2
AC51,PEG-7.5	84.00	g-m	6.22	i1-y1	5.71	g1-p1	36.57	g1-e2	2.17	r1-k2
AC52,PEG-7.5	84.00	g-m	6.55	f1-t1	5.79	f1-p1	40.08	v-r1	2.48	e1-x1
AC53,PEG-7.5	84.00	g-m	6.80	c1-o1	5.31	n1-a2	36.93	g1-c2	3.09	k-c1
AC54,PEG-7.5	88.00	d-j	7.98	l-y	7.20	e-q	32.03	w1-t2	2.31	k1-d2
AC55,PEG-7.5	85.33	f-l	6.32	h1-x1	5.15	o1-b2	38.61	c1-z1	2.98	o-g1
AC56,PEG-7.5	84.00	g-m	6.60	f1-s1	5.87	c1-p1	38.13	d1-b2	2.77	v-o1
AC57,PEG-7.5	78.67	k-q	5.52	u1-i2	5.56	j1-u1	35.95	i1-i2	2.89	r-j1
AC58,PEG-7.5	89.33	c-i	4.70	h2-u2	4.04	f2-t2	37.97	d1-b2	2.65	z-s1
AC59,PEG-7.5	86.67	e-k	5.94	n1-d2	5.55	j1-u1	37.10	e1-c2	3.05	m-d1
AC60,PEG-7.5	93.33	a-f	6.85	b1-n1	5.80	e1-p1	42.33	q-m1	2.32	k1-d2
AC61,PEG-7.5	97.33	abc	7.20	v-h1	6.80	i-z	31.40	a2-u2	1.67	k2-r2
AC62,PEG-7.5	89.33	c-i	7.25	v-h1	6.78	i-z	36.19	h1-h2	2.54	c1-v1
AC63,PEG-7.5	93.33	a-f	7.10	x-i1	6.81	i-z	34.15	q1-m2	2.29	k1-e2
AC64,PEG-7.5	94.67	a-e	5.58	u1-i2	4.58	y1-l2	35.87	i1-i2	2.49	d1-w1
AC1,PEG-15	91.67	b-g	3.58	y2-j3	2.70	a3-c3	28.87	i2-z2	4.12	bc
AC2,PEG-15	83.33	h-m	4.29	n2-a3	3.46	r2-a3	23.82	v2-b3	2.75	x-q1
AC3,PEG-15	68.33	stu	2.74	i3-l3	2.96	y2-c3	28.02	l2-z2	3.53	d-o
AC4,PEG-15	90.00	c-h	2.82	h3-l3	1.83	d3e3	32.68	s1-r2	2.93	p-h1
AC5,PEG-15	81.67	i-n	3.09	f3-l3	2.43	c3d3	16.72	b3	2.43	g1-z1
AC6,PEG-15	91.67	b-g	3.11	e3-k3	3.05	w2-c3	26.58	p2-z2	3.35	f-u

AC7,PEG-15	80.00 j-p	4.41 m2-z2	3.80 l2-w2	28.90 i2-z2	2.80 t-o1
AC8,PEG-15	46.67 y	2.01 m3	1.36 e3f3	34.12 q1-m2	5.00 a
AC9,PEG-15	90.00 c-h	5.63 t1-h2	4.07 f2-t2	34.18 p1-m2	3.48 d-q
AC10,PEG-15	83.33 h-m	3.86 t2-f3	3.61 n2-y2	33.22 r1-r2	4.42 b
AC11,PEG-15	46.67 y	3.85 u2-f3	3.81 k2-w2	24.52 u2-a3	1.80 d2-q2
AC12,PEG-15	85.00 g-l	6.01 k1-c2	4.45 b2-m2	28.93 i2-z2	3.03 n-e1
AC13,PEG-15	20.00 z	0.83 n3	1.00 F3	17.55 a3b3	3.10 k-c1
AC14,PEG-15	76.67 m-r	3.68 w2-i3	3.43 r2-a3	25.28 t2-z2	2.93 p-h1
AC15,PEG-15	88.33 d-i	5.96 l1-c2	3.05 w2-c3	36.40 g1-f2	3.62 c-l
AC16,PEG-15	78.33 l-q	3.65 w2-j3	4.16 d2-s2	28.37 k2-z2	2.20 p1-i2
AC17,PEG-15	81.67 i-n	5.93 n1-e2	2.95 y2-c3	33.73 r1-o2	3.31 f-x
AC18,PEG-15	81.67 i-n	5.17 a2-n2	3.43 s2-a3	37.97 d1-b2	3.83 c-g
AC19,PEG-15	71.67 q-t	6.32 h1-x1	3.28 t2-b3	37.01 f1-c2	3.99 bcd
AC20,PEG-15	75.00 n-s	6.03 k1-b2	3.88 j2-v2	38.32 d1-b2	4.42 b
AC21,PEG-15	78.33 l-q	4.71 h2-u2	2.62 b3c3	32.58 t1-s2	3.06 l-c1
AC22,PEG-15	78.33 l-q	5.15 a2-p2	3.28 t2-b3	28.59 j2-z2	2.99 o-g1
AC23,PEG-15	85.00 g-l	4.64 i2-w2	3.42 s2-a3	27.79 l2-z2	2.54 c1-v1
AC24,PEG-15	83.33 h-m	5.23 z1-m2	3.49 p2-a3	27.89 l2-z2	2.99 o-g1
AC25,PEG-15	81.67 i-n	5.06 c2-r2	3.92 i2-u2	38.31 d1-b2	3.37 e-s
AC26,PEG-15	90.00 c-h	3.21 b3-j3	4.00 g2-t2	23.71 w2-b3	2.48 e1-x1
AC27,PEG-15	91.67 b-g	3.20 b3-j3	3.49 p2-a3	22.44 y2-b3	2.57 c1-u1
AC28,PEG-15	86.67 e-k	5.22 z1-n2	3.47 q2-a3	36.79 g1-d2	3.61 c-m
AC29,PEG-15	85.00 g-l	5.09 b2-q2	3.89 j2-v2	34.08 q1-n2	3.37 e-s
AC30,PEG-15	53.33 xy	3.72 w2-h3	2.94 y2-c3	30.70 c2-w2	4.01 bcd
AC31,PEG-15	88.33 d-i	4.99 d2-s2	3.29 t2-b3	30.85 c2-v2	3.19 h-z
AC32,PEG-15	86.67 e-k	4.81 g2-u2	3.60 n2-y2	29.21 g2-y2	3.14 j-b1
AC33,PEG-15	60.00 vwx	3.32 b3-j3	3.07 w2-c3	33.83 r1-o2	3.00 o-f1
AC34,PEG-15	85.33 f-l	4.92 f2-s2	3.60 n2-y2	28.65 j2-z2	2.33 j1-d2
AC35,PEG-15	66.67 tuv	3.47 z2-j3	3.11 v2-c3	30.30 c2-x2	2.78 v-o1
AC36,PEG-15	81.33 i-o	4.91 f2-s2	3.87 j2-v2	35.23 m1-j2	3.69 c-j
AC37,PEG-15	62.67 uvw	3.64 x2-j3	2.76 z2-c3	31.23 b2-u2	3.69 c-j
AC38,PEG-15	90.67 b-h	4.08 r2-d3	3.07 w2-c3	26.15 r2-z2	2.79 u-o1
AC39,PEG-15	90.67 b-h	5.15 a2-o2	2.93 y2-c3	26.87 o2-z2	2.81 s-n1
AC40,PEG-15	78.67 k-q	4.68 h2-v2	3.78 l2-x2	36.37 g1-f2	3.36 e-t
AC41,PEG-15	86.67 e-k	4.02 s2-e3	3.53 n2-z2	27.96 l2-z2	2.32 k1-d2
AC42,PEG-15	85.33 f-l	4.11 q2-b3	4.07 f2-t2	22.09 z2-b3	1.69 i2-r2
AC43,PEG-15	86.67 e-k	5.44 x1-l2	4.26 d2-q2	23.37 x2-b3	2.26 n1-h2
AC44,PEG-15	72.00 p-t	3.82 v2-g3	3.17 u2-c3	35.55 k1-j2	3.32 f-w
AC45,PEG-15	80.00 j-p	5.17 a2-n2	5.15 o1-b2	33.05 r1-r2	3.13 j-b1
AC46,PEG-15	84.00 g-m	4.11 r2-c3	4.01 g2-t2	32.54 t1-s2	2.84 r-l1
AC47,PEG-15	80.00 j-p	4.19 p2-a3	4.31 c2-o2	26.99 n2-z2	2.03 u1-m2
AC48,PEG-15	76.00 m-s	4.53 l2-y2	4.31 c2-o2	25.47 s2-z2	1.99 v1-n2
AC49,PEG-15	81.33 i-o	4.07 s2-e3	3.52 o2-z2	31.96 x1-t2	3.09 k-c1
AC50,PEG-15	86.67 e-k	7.24 v-h1	4.23 d2-p2	29.10 h2-z2	2.91 r-i1
AC51,PEG-15	81.33 i-o	2.69 k3l3	3.87 j2-v2	33.43 r1-p2	3.39 e-r
AC52,PEG-15	81.33 i-o	4.65 i1-v2	3.69 m2-y2	31.53 z1-u2	3.27 g-y
AC53,PEG-15	85.33 f-l	6.37 h1-x1	4.92 q1-d2	32.23 v1-t2	3.63 c-k
AC54,PEG-15	90.67 b-h	7.45 t-f1	4.70 w1-i2	29.52 e2-y2	3.33 f-v
AC55,PEG-15	86.67 e-k	7.22 v-h1	4.62 x1-j2	32.05 v1-t2	2.29 k1-e2
AC56,PEG-15	84.00 g-m	8.02 k-y	4.77 u1-g2	34.87 o1-l2	2.04 u1-m2
AC57,PEG-15	77.33 l-r	5.10 a2-p2	4.50 b2-l2	30.79 c2-w2	3.39 e-r
AC58,PEG-15	90.67 b-h	3.18 c3-k3	3.47 q2-a3	32.24 u1-t2	3.07 k-c1
AC59,PEG-15	77.33 l-r	5.74 q1-g2	4.44 b2-m2	27.26 m2-z2	3.07 k-c1
AC60,PEG-15	78.67 k-q	5.33 y1-l2	4.86 t1-f2	38.39 c1-a2	3.83 c-f
AC61,PEG-15	94.67 a-e	6.34 h1-x1	4.42 b2-m2	26.13 r2-z2	2.15 s1-l2
AC62,PEG-15	80.00 j-p	6.65 e1-r1	4.43 b2-m2	29.45 f2-y2	3.59 c-n
AC63,PEG-15	92.00 a-g	6.06 j1-a2	6.07 y-n1	26.72 o2-z2	2.29 k1-e2
AC64,PEG-15	88.00 d-j	4.02 t2-f3	3.00 x2-c3	36.33 h1-f2	3.15 i-a1

GP: germination percentage, RL: root length, SL: shoot length, FW: fresh weight, DW: dry weight. Different letters denote a statistically significant variation in mean values measured by Duncan's Multiple-Range Test ($p \leq 0.01$).

Table S6. The mean values of biochemical traits under control (0% PEG) condition of drought.

Accessions	PC		SSC		TPC		AC		GPA		CAT		LP	
AC1	268.87	ai	104.07	t-aa	84.23	h-k	587.57	i-l	0.218	d-j	103.90	b-h	4.39	r-u
AC2	688.87	x-ab	128.15	n-r	89.85	fgh	599.73	e-i	0.186	f-m	25.97	m	3.73	z-ac
AC3	798.36	r-v	140.49	k-n	79.74	h-o	634.86	bc	0.129	k-n	45.45	j-m	4.44	r-u
AC4	487.85	ae-af	106.85	s-z	127.87	ab	500.41	abac	0.226	d-i	32.47	lm	3.79	y-ab
AC5	602.72	ab-ad	114.88	q-w	70.00	o-v	599.73	e-i	0.250	a-g	45.45	j-m	6.82	a
AC6	376.56	agah	124.75	n-s	131.80	a	486.89	acad	0.226	d-i	149.35	a	5.95	cd
AC7	313.74	ahai	121.67	n-u	98.28	efg	640.27	ab	0.323	ab	90.91	d-i	3.53	ab-af
AC8	696.05	y-ab	66.42	ad	98.84	efg	592.97	g-j	0.137	j-n	123.38	a-e	4.40	r-u
AC9	678.10	y-ab	139.57	k-o	111.39	cd	541.62	p-v	0.089	n	97.40	c-i	4.63	p-s
AC10	983.23	j-m	140.80	k-n	86.67	hij	613.24	de	0.129	k-n	97.40	c-i	4.94	j-p
AC11	466.31	ae-ag	103.15	u-aa	60.26	u-ab	653.78	a	0.194	e-m	38.96	klm	3.34	ae-af
AC12	681.69	y-ab	137.10	m-p	88.54	ghi	548.38	o-t	0.274	a-e	84.42	e-j	5.24	g-j
AC13	651.18	a-b	110.56	r-x	65.69	q-x	530.14	u-y	0.202	d-l	71.43	g-l	4.24	t-x
AC14	767.85	t-z	190.19	b-e	118.13	bc	540.95	p-v	0.234	c-i	149.35	a	3.68	aa-ad
AC15	1144.77	e-h	158.09	g-l	124.31	ab	553.78	opq	0.121	lmn	64.94	h-m	5.40	fg
AC16	857.59	n-t	108.70	r-y	104.08	de	556.49	nop	0.113	mn	116.88	a-f	4.24	t-x
AC17	1121.44	f-i	154.07	i-m	83.11	h-m	531.49	t-y	0.186	f-m	103.90	b-h	4.19	u-x
AC18	1388.87	b	85.56	aa-ad	81.61	h-n	601.08	e-i	0.161	h-n	123.38	a-e	2.98	ag
AC19	1135.79	e-h	94.81	x-ac	80.49	h-o	601.76	e-i	0.089	n	149.35	a	4.61	qrs
AC20	925.79	k-o	120.43	o-u	70.00	o-v	605.14	d-h	0.169	g-n	90.91	d-i	3.29	af
AC21	916.82	l-p	180.62	c-f	89.29	gh	621.35	cd	0.169	g-n	123.38	a-e	3.61	a-e
AC22	1263.23	cd	173.52	e-i	75.24	k-r	602.43	e-i	0.186	f-m	84.42	e-j	4.69	n-r
AC23	909.64	l-q	113.33	q-x	51.84	z-ab	590.95	h-k	0.282	a-d	103.90	b-h	5.60	ef
AC24	764.26	t-z	183.09	b-f	64.38	r-y	570.68	mn	0.161	h-n	110.39	a-g	4.47	r-u
AC25	1065.79	g-j	118.89	p-u	59.33	v-ab	609.19	d-g	0.266	a-f	142.86	ab	5.29	ghi
AC26	916.82	l-p	180.62	c-f	77.87	i-p	532.16	s-y	0.266	a-f	123.38	a-e	5.08	h-m
AC27	810.92	q-v	128.46	n-r	77.30	j-p	486.22	acad	0.234	c-i	71.43	g-l	3.38	ad-af
AC28	1055.03	hjj	171.05	e-j	49.96	ab	552.43	o-r	0.242	b-h	71.43	g-l	6.19	bc
AC29	545.28	ac-ae	175.37	d-h	63.45	s-y	546.35	o-u	0.202	d-l	149.35	a	5.94	cd
AC30	791.18	s-w	159.01	g-k	68.31	p-w	599.73	e-i	0.226	d-i	97.40	c-i	4.52	rst
AC31	785.79	s-x	158.09	g-l	70.94	n-u	515.27	y-ab	0.210	d-k	149.35	a	4.89	l-q
AC32	1320.67	bc	132.78	n-q	80.30	h-o	520.68	x-aa	0.210	d-k	58.44	i-m	4.65	o-s
AC33	839.64	n-u	96.98	v-ab	63.82	s-y	573.38	lm	0.210	d-k	136.36	abc	3.31	ae-af
AC34	518.36	ad-af	79.38	aa-ad	83.48	h-l	563.24	mno	0.202	d-l	38.96	klm	3.61	ab-ae
AC35	764.26	t-z	156.23	h-m	74.12	k-s	549.05	o-s	0.186	f-m	116.88	a-f	5.23	ghij
AC36	778.62	s-y	104.38	t-aa	81.99	h-n	601.76	e-i	0.137	j-n	97.40	c-i	5.92	cd
AC37	1123.23	f-i	154.38	i-m	62.70	t-z	595.68	f-i	0.218	d-j	71.43	g-l	5.21	g-k
AC38	825.28	o-v	106.23	s-z	58.58	w-ab	546.35	o-u	0.153	i-n	58.44	i-m	5.37	fgh
AC39	1403.23	b	138.95	l-o	89.66	fgh	526.08	v-z	0.169	g-n	58.44	i-m	3.95	x-aa
AC40	936.56	k-n	108.70	r-y	59.33	v-ab	550.41	o-r	0.121	lmn	129.87	a-d	4.35	s-v
AC41	731.95	v-aa	95.12	w-ac	54.64	x-ab	529.46	u-y	0.161	h-n	90.91	d-i	4.03	w-z
AC42	1697.59	a	214.57	a	67.57	p-w	540.27	p-v	0.282	a-d	71.43	g-l	3.39	ad-af
AC43	620.67	abac	67.96	ad	62.51	t-aa	519.32	x-aa	0.178	g-m	71.43	g-l	3.84	y-ab
AC44	880.92	n-s	176.30	d-g	79.93	h-o	605.14	d-h	0.202	d-l	71.43	g-l	4.60	qrs
AC45	439.38	af-ag	152.84	j-m	76.37	j-q	576.76	j-m	0.194	e-m	58.44	i-m	6.45	b
AC46	740.92	u-aa	88.64	z-ac	81.24	h-o	535.54	r-x	0.153	i-n	84.42	e-j	5.76	de
AC47	845.03	n-t	104.38	t-aa	72.25	l-t	470.68	adae	0.274	a-e	90.91	d-i	3.37	ad-af
AC48	1191.44	def	166.11	f-j	58.39	w-ab	575.41	klm	0.323	ab	90.91	d-i	3.73	z-ac
AC49	1159.13	efg	160.56	g-j	82.36	h-m	611.89	def	0.250	a-g	71.43	g-l	5.24	g-j
AC50	801.95	r-v	160.86	g-j	105.58	de	508.51	aa-ab	0.331	a	71.43	g-l	3.32	aeaf
AC51	796.56	r-w	103.15	u-aa	53.33	y-ab	576.08	klm	0.194	e-m	77.92	f-k	4.95	j-o
AC52	1406.82	b	201.30	ab	63.82	s-y	578.78	j-m	0.153	i-n	64.94	h-m	4.98	i-n
AC53	1024.51	ijk	75.68	ac-ad	51.09	ab	538.24	q-w	0.121	lmn	77.92	f-k	4.27	t-w
AC54	496.82	ae-af	107.16	s-z	88.91	ghi	486.22	acad	0.331	a	77.92	f-k	6.37	b
AC55	927.59	k-n	123.21	n-t	51.46	aaab	551.08	o-r	0.169	g-n	77.92	f-k	3.94	x-aa
AC56	543.49	ac-ae	90.56	y-ac	80.86	h-o	548.38	o-t	0.153	i-n	116.88	a-f	4.90	k-q
AC57	676.31	z-ab	77.53	ab-ad	77.12	j-p	603.11	e-i	0.218	d-j	64.94	h-m	5.21	g-l
AC58	645.79	aaab	195.74	bc	59.14	v-ab	472.03	adae	0.169	g-n	58.44	i-m	3.32	aeaf
AC59	819.90	p-v	104.69	t-aa	100.15	ef	553.78	opq	0.274	a-e	149.35	a	4.84	m-q
AC60	895.28	m-r	115.19	q-v	50.71	ab	461.89	ae	0.194	e-m	58.44	i-l	3.81	y-ab
AC61	1006.56	j-l	192.96	bcd	88.73	ghi	522.03	w-aa	0.218	d-j	71.43	g-l	5.19	g-l
AC62	674.51	z-ab	77.22	ac-ad	72.06	m-t	510.54	z-ab	0.153	i-n	84.42	e-j	3.44	ac-af
AC63	1064.00	g-j	85.56	aa-ad	89.48	fgh	521.35	w-aa	0.274	a-e	71.43	g-l	4.05	v-y
AC64	1227.33	cde	110.56	r-x	88.91	ghi	530.14	u-y	0.202	d-l	64.94	h-m	4.90	k-q

PC: proline content, SSC: soluble sugar content, TPC: total phenolic content, AC: antioxidant activity, GPA: guaiacol peroxidase activity, CAT: catalase activity, LP: lipid peroxidation. Different letters denote a statistically significant variation in mean values measured by Duncan's Multiple-Range Test ($p \leq 0.01$).

Table S7. The mean values of biochemical traits under 7.5% PEG-induced condition.

Accessions	PC		SSC		TPC		AC		GPA		CAT		LP	
AC1	841.44	afag	130.62	adae	225.24	yz	697.03	e	0.420	d-g	149.35	nop	6.19	lm
AC2	739.13	ahai	167.96	u	259.70	t	664.59	m-q	0.347	k-o	84.42	u	4.90	aa
AC3	897.08	aeaf	186.79	pqr	234.98	wx	717.30	b	0.266	u	142.86	opq	5.37	vw
AC4	1042.46	ac	154.69	wxy	398.09	f	674.05	jk	0.428	def	175.32	jkl	4.06	ag
AC5	1295.54	wx	178.15	st	242.85	v	663.92	n-q	0.339	l-p	142.86	opq	7.79	b
AC6	631.44	aj	181.54	rst	403.52	e	695.68	e	0.428	def	227.27	cd	7.13	ef
AC7	638.62	aj	145.43	z-ab	225.62	yz	719.32	b	0.371	i-l	149.35	nop	4.60	acad
AC8	794.77	agai	141.11	abac	133.11	ak	642.30	uv	0.186	w	162.34	lmn	6.71	j
AC9	2270.15	f	167.04	u	312.13	n	722.70	b	0.347	k-o	279.22	b	5.31	wx
AC10	1205.79	yz	147.28	z-ab	166.63	agah	679.46	g-j	0.202	vw	162.34	lmn	6.42	k
AC11	617.08	aj	146.36	z-ab	92.85	al	736.89	a	0.274	tu	129.87	qr	5.47	uv
AC12	1345.79	uvw	144.20	z-ab	214.01	ab	651.08	t	0.387	g-j	162.34	lmn	6.71	j
AC13	726.56	ai	136.17	acad	139.10	aj	610.54	z	0.266	u	90.91	u	6.63	j
AC14	845.03	afag	218.58	k	198.84	adae	559.19	ab	0.315	o-s	188.31	hij	5.15	z
AC15	1991.95	i	230.82	ij	214.57	ab	613.85	yz	0.420	d-g	194.81	ghi	6.47	k
AC16	1067.59	abac	181.23	rst	185.54	af	584.86	aa	0.379	h-k	188.31	hij	5.55	tu
AC17	1701.18	no	189.57	opq	220.37	zaa	636.89	v	0.323	n-r	188.31	hij	5.84	qr
AC18	1668.87	op	125.99	ae	170.75	ag	688.92	f	0.282	stu	149.35	nop	5.23	xyz
AC19	1376.31	tuv	131.23	adae	154.83	ai	654.46	st	0.218	v	188.31	hij	6.21	lm
AC20	1019.13	ac	195.74	mno	164.01	ah	645.00	u	0.282	stu	162.34	lmn	5.27	wxy
AC21	1304.51	wx	244.51	fg	204.46	ac	697.03	e	0.307	p-t	188.31	hij	5.32	wx
AC22	1711.95	no	219.20	k	194.16	ae	706.49	cd	0.323	n-r	168.83	klm	6.06	no
AC23	1430.15	st	219.51	k	201.09	acad	677.43	ij	0.428	def	175.32	jkl	7.31	d
AC24	1317.08	vw	233.09	ij	248.46	u	738.92	a	0.323	n-r	214.29	def	5.73	rs
AC25	1157.33	zaa	241.42	hi	220.75	zaa	662.57	opq	0.371	i-l	201.30	fgh	6.90	i
AC26	2462.21	e	305.93	b	332.73	l	684.86	fg	0.379	h-k	233.77	c	5.92	pq
AC27	2205.54	g	258.09	e	366.25	h	665.27	m-p	0.476	bc	220.78	cde	3.77	ai
AC28	2521.44	d	276.60	c	290.22	p	678.11	hij	0.395	f-i	220.78	cde	7.16	e
AC29	1119.64	aaab	227.84	j	272.43	r	668.65	k-n	0.307	p-t	188.31	hij	7.47	c
AC30	751.69	ahai	166.11	u	149.78	ai	684.86	fg	0.339	l-p	149.35	nop	6.66	j
AC31	1611.44	pq	196.36	mn	339.10	k	641.62	uv	0.363	i-m	214.29	def	5.18	yz
AC32	1918.36	j	229.69	ij	358.39	i	631.49	w	0.420	d-g	201.30	fgh	5.29	wxy
AC33	1011.95	acad	126.30	ae	183.67	af	659.86	pqrs	0.282	stu	214.29	def	5.45	uv
AC34	1738.87	mn	156.85	wx	311.01	n	656.49	rst	0.444	de	175.32	jkl	4.60	acad
AC35	1187.85	yz	146.98	z-ab	171.31	ag	643.65	u	0.266	u	155.84	mno	6.95	hi
AC36	1317.08	vw	134.01	ad	219.06	aaab	666.62	l-o	0.299	q-u	162.34	lmn	7.10	efg
AC37	1209.38	yz	200.68	lm	149.78	ai	697.03	e	0.331	m-q	149.35	nop	7.08	efg
AC38	2072.72	h	204.38	l	336.67	kl	640.95	uv	0.355	j-n	188.31	hij	5.63	st
AC39	2643.49	b	309.94	b	370.94	h	698.38	e	0.387	g-j	188.31	hij	4.76	ab
AC40	1381.69	stu	149.14	yz	237.98	vw	659.19	qrs	0.379	h-k	149.35	nop	5.82	qr
AC41	1412.21	st	191.73	nop	326.93	m	655.81	rst	0.363	i-m	149.35	nop	4.79	ab
AC42	2458.62	e	233.40	ij	395.84	f	680.14	ghi	0.452	cd	181.82	ijk	3.90	ah
AC43	1414.00	st	192.96	nop	421.87	d	688.92	f	0.290	r-u	168.83	klm	4.52	ad-af
AC44	1040.67	ac	214.57	k	205.77	ac	664.59	m-q	0.387	g-j	110.39	st	5.55	tu
AC45	1078.36	abac	248.21	f	249.96	u	684.19	fg	0.282	stu	97.40	tu	7.97	a
AC46	1699.38	no	177.84	st	379.74	g	671.35	kl	0.347	k-o	162.34	lmn	6.11	mn
AC47	2108.62	h	141.73	aa-ac	396.03	f	616.62	xy	0.411	e-h	168.83	klm	3.87	ahai
AC48	1819.64	kl	235.56	ij	259.14	t	671.35	kl	0.379	h-k	136.36	pqr	4.45	aeaf
AC49	1444.51	s	159.01	vw	183.11	af	670.00	klm	0.355	j-n	162.34	lmn	7.03	fgh
AC50	1842.97	k	204.69	l	282.73	q	588.24	aa	0.508	a	168.83	klm	4.42	af
AC51	1245.28	xy	167.35	u	229.55	xy	683.51	fgh	0.323	n-r	162.34	lmn	7.00	ghi
AC52	1539.64	r	189.88	n-q	241.54	v	710.54	c	0.379	h-k	123.38	rs	6.13	mn
AC53	1537.85	r	150.68	xyz	265.51	s	719.32	b	0.299	q-u	136.36	pqr	5.28	wxy
AC54	1776.56	lm	213.95	k	431.24	c	611.89	yz	0.500	ab	207.79	efg	7.08	efg
AC55	1399.64	stu	141.11	abac	400.71	ef	703.78	d	0.347	k-o	162.34	lmn	4.92	aa
AC56	1602.46	q	180.31	rst	341.91	jk	667.30	l-o	0.339	l-p	175.32	jkl	5.63	st
AC57	929.38	ae	124.75	ae	230.30	xy	682.84	ghi	0.331	m-q	194.81	ghi	7.11	efg
AC58	954.51	adae	339.88	a	345.09	j	534.19	ac	0.323	n-r	175.32	jkl	4.76	ab
AC59	1072.97	abac	163.95	uv	233.48	wx	651.76	t	0.363	i-m	272.73	b	6.31	l
AC60	1042.46	ac	158.09	vw	340.41	jk	615.27	yz	0.331	m-q	155.84	mno	4.68	abac
AC61	2961.18	a	264.57	d	505.58	a	660.54	pqr	0.363	i-m	305.19	a	5.84	qr
AC62	1279.38	wx	176.91	t	289.66	p	640.95	uv	0.307	p-t	162.34	lmn	4.55	adae
AC63	2582.46	c	148.52	y-aa	453.15	b	651.08	t	0.420	d-g	181.82	ijk	4.63	acad
AC64	1597.08	qr	184.01	qrs	301.46	o	620.68	x	0.347	k-o	149.35	nop	5.98	op

PC: proline content, SSC: soluble sugar content, TPC: total phenolic content, AC: antioxidant activity, GPA: guaiacol peroxidase activity, CAT: catalase activity, LP: lipid peroxidation. Different letters denote a statistically significant variation in mean values measured by Duncan's Multiple-Range Test ($p \leq 0.01$).

Table S8. The mean values of biochemical traits under 15%PEG-induced condition.

Accessions	PC		SSC		TPC		AC		GPA		CAT		LP	
AC1	1340.41	ab	201.30	uv	317.75	ae	696.35	wxy	0.331	s-v	136.36	pqr	8.13	cd
AC2	952.72	af	226.91	o	374.49	x	686.22	y	0.266	y-aa	97.40	u-x	5.69	xy
AC3	1058.62	ae	203.46	tu	341.16	ab	741.62	o-r	0.250	z-ab	116.88	r-u	6.16	qrs
AC4	3386.56	i	311.17	e	514.76	i	791.62	f-i	0.541	fgh	311.69	b	4.31	ag
AC5	1765.79	v	301.60	f	352.21	aa	686.22	y	0.363	o-r	110.39	s-v	9.19	a
AC6	3241.18	k	271.36	ij	542.28	g	839.59	a	0.589	bcd	266.23	e	6.21	qr
AC7	1604.26	x	172.90	z	380.67	w	767.97	j-n	0.371	n-q	181.82	klm	4.95	ad
AC8	904.26	ag	192.04	wx	225.24	ao	632.16	aa	0.234	abac	155.84	nop	8.40	b
AC9	4607.08	b	286.79	h	553.33	f	834.86	a	0.581	cde	77.92	xy	5.00	acad
AC10	1755.03	v	298.52	fg	327.12	ad	736.89	pqr	0.282	xy	149.35	opq	7.85	e
AC11	841.44	ahai	129.69	ae	189.10	aq	734.86	qr	0.234	abac	84.42	wxy	6.10	rs
AC12	2271.95	r	385.56	b	364.76	y	754.46	l-p	0.452	klm	175.32	lmn	7.13	l
AC13	857.59	ah	152.53	ac	210.26	ap	611.89	ab	0.242	aa-ac	71.43	y	8.00	de
AC14	1234.51	ac	156.23	abac	262.13	am	570.68	ac	0.282	xy	149.35	opq	6.13	rs
AC15	2914.51	l	266.11	jk	373.75	x	712.57	s-w	0.323	tuv	155.84	nop	7.15	l
AC16	1548.62	yz	196.67	vw	289.10	ai	665.27	z	0.347	q-t	149.35	opq	6.53	no
AC17	2246.82	r	208.70	st	334.23	ac	655.14	z	0.379	nop	136.36	pqr	6.05	r-u
AC18	2126.56	st	130.93	ae	293.97	ah	686.22	y	0.323	tuv	136.36	pqr	6.06	rst
AC19	1914.77	u	96.36	ag	240.04	an	660.54	z	0.218	ac	181.82	klm	7.18	kl
AC20	1266.82	ac	134.63	adae	266.63	al	654.46	z	0.290	wxy	116.88	r-u	6.08	rs
AC21	1658.10	w	217.65	qr	275.24	ajak	716.62	stu	0.234	abac	181.82	klm	5.89	vw
AC22	2124.77	st	236.79	n	279.36	aj	735.54	qr	0.387	no	123.38	rst	6.37	p
AC23	2361.69	q	220.43	pq	308.58	af	736.89	pqr	0.524	hi	168.83	mno	7.69	f
AC24	2148.10	s	251.91	m	380.86	w	725.41	rst	0.379	nop	188.31	j-m	6.05	r-u
AC25	1593.49	xy	209.32	st	279.36	aj	697.70	v-y	0.331	s-v	194.81	i-l	7.26	kl
AC26	3783.23	g	393.27	a	486.10	m	774.73	ijk	0.524	hi	292.21	bcd	6.11	rs
AC27	4714.77	a	396.36	a	512.13	ij	793.65	e-h	0.605	bc	305.19	bc	4.18	ag
AC28	3379.38	i	299.14	fg	481.24	n	815.95	bc	0.468	kl	279.22	de	6.45	nop
AC29	1719.13	v	250.68	m	390.79	v	778.78	g-j	0.444	lm	188.31	j-m	7.52	gh
AC30	520.15	ak	92.35	ag	273.00	ak	664.59	z	0.339	r-u	116.88	r-u	7.35	ij
AC31	3395.54	i	275.99	i	477.68	n	710.54	t-w	0.476	jk	292.21	bcd	5.21	ab
AC32	3323.74	j	313.33	e	459.70	o	758.51	k-o	0.500	ij	285.71	cd	5.60	yz
AC33	807.33	ai	120.12	af	279.18	aj	685.54	y	0.274	yz	149.35	opq	6.00	s-v
AC34	2433.49	p	230.00	o	414.76	s	770.68	j-m	0.524	hi	201.30	ijk	5.55	yz
AC35	985.03	af	153.15	ac	288.73	ai	665.95	z	0.250	z-ab	136.36	pqr	8.10	cd
AC36	1478.62	aa	162.72	aa	305.77	afag	753.11	m-q	0.242	aa-ac	168.83	mno	8.24	c
AC37	1252.46	ac	164.26	aa	222.06	ao	689.59	xy	0.315	uvw	123.38	rst	8.47	b
AC38	3716.82	h	266.11	jk	504.08	k	724.73	rst	0.444	lm	233.77	fg	5.92	t-w
AC39	4312.72	c	293.27	g	563.45	d	825.41	ab	0.500	ij	292.21	bcd	5.13	abac
AC40	1624.00	wx	134.94	adae	314.01	ae	700.41	u-y	0.339	r-u	136.36	pqr	6.53	no
AC41	2279.13	r	152.22	ac	443.22	p	748.38	opq	0.468	kl	201.30	ijk	5.53	z
AC42	4021.95	e	303.15	f	558.76	e	807.84	c-f	0.557	efg	246.75	f	4.55	af
AC43	3352.46	ij	253.15	lm	575.06	c	826.08	ab	0.533	gh	227.27	gh	4.87	adae
AC44	1510.92	zaa	214.26	rs	330.30	ad	695.00	wxy	0.307	vwx	103.90	t-w	6.60	n
AC45	2329.38	q	199.75	uv	402.96	t	754.46	l-p	0.379	nop	90.91	v-y	8.16	c
AC46	2485.54	o	255.62	lm	522.62	h	795.00	d-g	0.452	klm	207.79	hij	6.31	pq
AC47	3377.59	i	180.31	y	544.72	g	715.27	s-v	0.565	def	214.29	ghi	4.74	ae
AC48	2088.87	t	195.43	vw	376.37	x	771.35	jkl	0.428	m	103.90	t-w	5.77	wx
AC49	1128.62	ad	171.36	z	302.40	ag	698.38	c-y	0.347	q-t	90.91	v-y	6.56	n
AC50	3207.08	k	264.26	k	490.22	l	725.41	rst	0.613	b	214.29	ghi	4.56	af
AC51	1349.38	ab	176.60	yz	357.27	z	776.76	hij	0.242	aa-ac	129.87	qrs	7.92	e
AC52	1905.79	u	224.75	op	349.78	aa	742.97	o-r	0.339	r-u	103.90	t-w	6.58	n
AC53	2672.21	m	199.14	uv	422.81	r	811.22	bcd	0.444	lm	168.83	mno	5.69	xy
AC54	3971.69	f	258.40	l	590.60	b	728.78	rs	0.654	a	298.70	bcd	7.31	ijk
AC55	2889.38	l	188.95	x	510.64	j	771.35	jkl	0.452	klm	214.29	ghi	5.84	wx
AC56	3237.59	k	115.19	af	390.97	v	751.76	n-q	0.452	klm	214.29	ghi	6.39	op
AC57	595.54	aj	84.32	ah	351.65	aa	708.51	t-w	0.339	r-u	123.38	rst	7.63	fg
AC58	2431.69	p	355.93	d	397.15	u	655.14	z	0.452	klm	168.83	mno	5.90	uvw
AC59	1543.23	z	139.57	ad	342.28	ab	697.70	v-y	0.355	p-s	181.82	klm	7.45	hi
AC60	1756.82	v	224.75	op	435.54	q	705.14	u-x	0.371	n-q	103.90	t-w	5.55	yz
AC61	4289.38	c	374.14	c	617.19	a	833.51	a	0.589	bcd	350.65	a	5.79	wx
AC62	2672.21	m	188.95	x	402.58	t	740.95	o-r	0.395	n	188.31	j-m	5.39	aa
AC63	4100.92	d	250.99	m	591.35	b	810.54	b-e	0.524	hi	285.71	cd	4.97	ad
AC64	2566.31	n	159.94	aaab	395.84	u	751.08	n-q	0.395	n	188.31	j-m	6.89	m

PC: proline content, SSC: soluble sugar content, TPC: total phenolic content, AC: antioxidant activity, GPA: guaiacol peroxidase activity, CAT: catalase activity, LP: lipid peroxidation. Different letters denote a statistically significant variation in mean values measured by Duncan's Multiple-Range Test ($p \leq 0.01$).

Table S9. The mean values of biochemical traits of all accessions under all drought conditions.

Accessions	PC		SCC		TPC		AC		GPA		CAT		LP	
AC1	816.91	g1	145.33	za1	209.08	c1	660.32	m-p	0.323	lmn	129.87	v-y	6.24	no
AC2	793.57	g1	174.34	rs	241.35	t	650.18	r-u	0.266	tu	69.26	g1	4.77	b1
AC3	918.02	d1	176.91	r	218.63	a1	697.93	b	0.215	za1	101.73	b1-e1	5.32	v
AC4	1638.96	o	190.91	op	346.90	f	655.36	o-s	0.398	ef	173.16	f-i	4.05	h1i1
AC5	1221.35	vw	198.21	klm	221.69	yz	649.95	r-u	0.317	mno	99.57	c1-e1	7.94	a
AC6	1416.39	r	192.55	no	359.20	d	674.05	g-j	0.414	cde	214.29	b	6.43	ij
AC7	852.21	f1	146.67	yz	234.86	v	709.19	a	0.353	ij	140.69	r-u	4.36	g1
AC8	798.36	g1	133.19	d1e1	152.40	n1	622.48	za1	0.186	b1	147.19	p-s	6.51	h
AC9	2518.44	d	197.80	lm	325.62	i	699.73	b	0.339	jkl	151.52	n-q	4.98	y
AC10	1314.68	t	195.53	mn	193.47	f1	676.53	e-i	0.204	a1	136.36	t-w	6.40	jk
AC11	641.61	j1	126.40	g1	114.07	q1	708.51	a	0.234	xy	84.42	f1	4.97	yz
AC12	1433.15	r	222.28	f	222.43	y	651.31	q-t	0.371	h	140.69	r-u	6.36	jkl
AC13	745.11	h1	133.09	d1e1	138.35	p1	584.19	f1	0.237	wxy	77.92	f1g1	6.29	lmn
AC14	949.13	c1	188.33	p	193.03	f1	556.94	g1	0.277	st	162.34	j-m	4.98	y
AC15	2017.08	j	218.34	g	237.54	u	626.73	yz	0.288	qrs	138.53	s-v	6.34	klm
AC16	1157.93	y	162.20	u	192.91	f1	602.21	c1d1	0.280	rst	151.52	n-q	5.44	t
AC17	1689.81	n	184.12	q	212.57	b1	607.84	b1c1	0.296	qr	142.86	q-u	5.36	uv
AC18	1728.10	m	114.16	h1	182.11	i1	658.74	nop	0.256	uv	136.36	t-w	4.76	b1
AC19	1475.62	q	107.47	i1	158.45	m1	638.92	vw	0.175	b1	173.16	f-i	6.00	p
AC20	1070.58	a1	150.27	wxy	166.88	k1	634.86	wx	0.247	vwx	123.38	yz	4.88	a1
AC21	1293.15	tu	214.26	h	189.66	g1	678.33	d-h	0.237	wxy	164.50	i-m	4.94	y-a1
AC22	1699.98	n	209.84	i	182.92	i1	681.49	def	0.299	pq	125.54	xy	5.71	r
AC23	1567.96	p	184.42	q	187.17	g1h1	668.42	jkl	0.411	de	149.35	o-r	6.87	e
AC24	1409.81	r	222.70	f	231.24	w	678.33	d-h	0.288	qrs	171.00	g-j	5.41	tu
AC25	1272.21	u	189.88	op	186.48	h1	656.49	opq	0.323	lmn	179.65	fg	6.48	hi
AC26	2387.42	e	293.27	b	298.90	k	663.92	lmn	0.390	fg	216.45	b	5.70	r
AC27	2577.08	c	260.97	d	318.56	j	648.38	tu	0.438	b	199.13	cd	3.78	k1
AC28	2318.62	f	248.93	e	273.81	n	682.16	de	0.368	hi	190.48	de	6.60	g
AC29	1128.02	z	217.96	gh	242.22	t	664.59	lmn	0.317	mno	175.32	fgh	6.97	d
AC30	687.68	i1	139.16	b1c1	163.70	l1	649.73	stu	0.301	opq	121.21	yz	6.18	o
AC31	1930.92	k	210.14	i	295.91	l	622.48	za1	0.350	jk	218.61	b	5.09	x
AC32	2187.59	g	225.27	f	299.46	k	636.89	w	0.377	gh	181.82	ef	5.18	w
AC33	886.31	e1	114.47	h1	175.56	j1	639.59	vw	0.256	uv	166.67	h-l	4.92	y-a1
AC34	1563.57	p	155.41	v	269.75	o	663.47	lmn	0.390	fg	138.53	s-v	4.59	d1e1
AC35	979.04	b1	152.12	vw	178.05	j1	619.55	a1	0.234	xy	136.36	t-w	6.76	f
AC36	1191.44	x	133.70	d1e1	202.27	e1	673.83	g-j	0.226	yz	142.86	q-u	7.09	c
AC37	1195.03	wx	173.11	s	144.84	o1	660.77	m-p	0.288	qrs	114.72	za1	6.92	de
AC38	2204.94	g	192.24	no	299.78	k	637.34	w	0.317	mno	160.17	k-n	5.64	rs
AC39	2786.48	a	247.39	e	341.35	g	683.29	d	0.352	ij	179.65	fg	4.61	c1-e1
AC40	1314.09	t	130.93	e1f1	203.77	d1e1	636.67	wx	0.280	rst	138.53	s-v	5.57	s
AC41	1474.43	q	146.36	z	274.93	n	644.55	uv	0.331	lmn	147.19	p-s	4.78	b1
AC42	2726.05	b	250.37	e	340.72	g	676.08	f-i	0.430	bc	166.67	h-l	3.95	j1
AC43	1795.71	l	171.36	s	353.15	e	678.11	d-h	0.333	klm	155.84	m-p	4.41	f1g1
AC44	1144.17	yz	201.71	jk	205.33	d1	654.91	p-s	0.299	pq	95.24	e1	5.58	s
AC45	1282.38	u	200.27	kl	243.10	t	671.80	ijk	0.285	qrs	82.25	f1	7.53	b
AC46	1641.95	o	174.03	rs	327.87	i	667.30	kl	0.317	mno	151.52	n-q	6.06	p
AC47	2110.41	h	142.14	a1b1	337.67	h	600.86	d1	0.417	cd	158.01	l-o	3.99	i1j1
AC48	1699.98	n	199.03	klm	231.30	w	672.70	h-k	0.377	gh	110.39	a1b1	4.65	c1d1
AC49	1244.09	v	163.64	tu	189.29	g1	660.09	m-p	0.317	mno	108.23	a1-c1	6.28	mn
AC50	1950.67	k	209.94	i	292.85	m	607.39	b1c1	0.484	a	151.52	n-q	4.10	h1
AC51	1130.41	yz	149.03	w-z	213.38	b1	678.78	d-g	0.253	uvw	123.38	yz	6.62	g
AC52	1617.42	o	205.31	j	218.38	a1	677.43	d-i	0.290	qrs	97.40	d1e1	5.90	q
AC53	1744.85	m	141.83	a1b1	246.47	s	689.59	c	0.288	qrs	127.71	wxy	5.08	x
AC54	2081.69	i	193.17	no	370.25	c	608.96	b1	0.495	a	194.81	cd	6.92	de
AC55	1738.87	m	151.09	wx	320.94	j	675.41	ghi	0.323	lmn	151.52	n-q	4.90	za1
AC56	1794.51	l	128.68	f1g1	271.25	o	655.81	o-r	0.315	nop	168.83	h-k	5.64	rs
AC57	733.74	h1	95.53	j1	219.69	za1	664.82	lm	0.296	qr	127.71	wxy	6.65	g
AC58	1344.00	s	297.18	a	267.13	p	553.78	g1	0.315	nop	134.20	u-x	4.66	c1
AC59	1145.37	yz	136.07	c1d1	225.31	x	634.41	wx	0.331	lmn	201.30	c	6.20	o
AC60	1231.52	v	166.01	t	275.56	n	594.10	e1	0.299	pq	106.06	a1-d1	4.68	c1
AC61	2752.38	b	277.22	c	403.83	a	672.03	ijk	0.390	fg	242.42	a	5.61	s
AC62	1542.03	p	147.70	xyz	254.77	r	630.81	xy	0.285	qrs	145.02	q-t	4.46	f1
AC63	2582.46	c	161.69	u	377.99	b	660.99	mno	0.406	def	179.65	fg	4.55	e1
AC64	1796.91	l	151.50	w	262.07	q	633.96	wx	0.315	nop	134.20	u-x	5.92	q

PC: proline content, SSC: soluble sugar content, TPC: total phenolic content, AC: antioxidant activity, GPA: guaiacol peroxidase activity, CAT: catalase activity, LP: lipid peroxidation. Different letters denote a statistically significant variation in mean values measured by Duncan's Multiple-Range Test ($p \leq 0.01$).

Table S10. The interaction values of biochemical traits among the tomato accessions and treatments.

Status	PC		SSC		TPC		AC		GPA		CAT		LP	
AC1,Control	268.87	v3	104.07	p3	84.23	c3-e3	587.57	q2-s2	0.218	j1-m1	103.90	a1-c1	4.39	w2-y2
AC2,Control	688.87	g3-j3	128.15	d3-g3	89.85	a3b3	599.73	m2-p2	0.186	n1-q1	25.97	k1	3.73	i3-j3
AC3,Control	798.36	x2-b3	140.49	r2-w2	79.74	f3-j3	634.86	b2-d2	0.129	t1u1	45.45	i1j1	4.44	u2-w2
AC4,Control	487.85	r3s3	106.85	o3p3	127.87	t2u2	500.41	n3	0.226	i1-l1	32.47	j1k1	3.79	g3-j3
AC5,Control	602.72	n3o3	114.88	j3-m3	70.00	n3-q3	599.73	m2-p2	0.250	g1-i1	45.45	i1j1	6.82	uv
AC6,Control	376.56	u3	124.75	f3-i3	131.80	s2t2	486.89	o3	0.226	i1-l1	149.35	tuv	5.95	k1-n1
AC7,Control	313.74	v3	121.67	h3i3	98.28	z2	640.27	b2-d2	0.317	z-c1	90.91	c1-e1	3.53	l3m3
AC8,Control	696.05	f3-i3	66.42	x3	98.84	z2	592.97	o2-r2	0.137	s1-u1	123.38	xyz	4.40	v2-x2
AC9,Control	678.10	i3-l3	139.57	s2-x2	111.39	w2	541.62	z2-c3	0.089	v1	97.40	b1-d1	4.63	o2-s2
AC10,Control	983.23	o2-q2	140.80	q2-w2	86.67	b3-d3	613.24	f2-j2	0.129	t1u1	97.40	b1-d1	4.94	g2-i2
AC11,Control	466.31	s3t3	103.15	p3q3	60.26	u3-w3	653.78	v1-y1	0.194	m1-p1	38.96	j1k1	3.34	n3o3
AC12,Control	681.69	h3-k3	137.10	u2-z2	88.54	a3-c3	548.38	y2-b3	0.274	e1-g1	84.42	d1-f1	5.24	a2-d2
AC13,Control	651.18	i3-m3	110.56	l3-o3	65.69	q3-t3	530.14	d3-i3	0.202	l1-o1	71.43	f1-h1	4.24	z2-b3
AC14,Control	767.85	a3-e3	190.19	l1-o1	118.13	v2	540.95	z2-c3	0.234	i1-k1	149.35	tuv	3.68	j3k3
AC15,Control	1144.77	f2g2	158.09	c2-i2	124.31	u2	553.78	w2-y2	0.121	u1	64.94	f1-h1	5.40	x1y1
AC16,Control	857.59	u2-w2	108.70	m3-p3	104.08	x2y2	556.49	w2-y2	0.113	u1v1	116.88	y-a1	4.24	z2-b3
AC17,Control	1121.44	g2-h2	154.07	g2-k2	83.11	d3-f3	531.49	c3-h3	0.186	n1-q1	103.90	a1-c1	4.19	a3b3
AC18,Control	1388.87	r1-t1	85.56	u3v3	81.61	e3-h3	601.08	l2-p2	0.161	q1-s1	123.38	xyz	2.98	p3
AC19,Control	1135.79	g2	94.81	r3-t3	80.49	e3-j3	601.76	k2-o2	0.089	v1	149.35	tuv	4.61	p2-s2
AC20,Control	925.79	r2-t2	120.43	h3-j3	70.00	n3-q3	605.14	h2-n2	0.169	p1-r1	90.91	c1-e1	3.29	o3
AC21,Control	916.82	r2-t2	180.62	q1-t1	89.29	a3b3	621.35	e2f2	0.169	p1-r1	123.38	xyz	3.61	k3l3
AC22,Control	1263.23	y1-b2	173.52	u1-w1	75.24	k3-m3	602.43	k2-o2	0.186	n1-q1	84.42	d1-f1	4.69	m2-p2
AC23,Control	912.03	r2-t2	113.33	k3-n3	51.84	y3z3	590.95	p2-r2	0.282	d1-f1	103.90	a1-c1	5.60	u1v1
AC24,Control	764.26	a3-e3	183.09	p1-s1	64.38	r3-u3	570.68	u2v2	0.161	q1-s1	110.39	z-b1	4.47	t2-w2
AC25,Control	1065.79	j2l2	118.89	i3-k3	59.33	v3w3	609.19	g2-m2	0.266	f1-h1	142.86	uvw	5.29	y1-c2
AC26,Control	916.82	r2-t2	180.62	q1-t1	77.87	g3-k3	532.16	c3-g3	0.266	f1-h1	123.38	xyz	5.08	e2f2
AC27,Control	810.92	w2-a3	128.46	c3-g3	77.30	h3-k3	486.22	o3	0.234	i1-k1	71.43	f1-h1	3.38	n3o3
AC28,Control	1055.03	j2-m2	171.05	v1-y1	49.96	z3	552.43	x2y2	0.242	h1-j1	71.43	f1-h1	6.19	e1-g1
AC29,Control	545.28	p3	175.37	t1-v1	63.45	s3-v3	546.35	y2-b3	0.202	l1-o1	149.35	tuv	5.94	l1-o1
AC30,Control	791.18	z2-b3	159.01	c2-h2	68.31	o3-r3	599.73	m2-p2	0.226	i1-l1	97.40	b1-d1	4.52	s2-v2
AC31,Control	785.79	z2-c3	158.09	c2-i2	70.94	m3-p3	515.27	k3-m3	0.210	k1-n1	149.35	tuv	4.89	g2-j2
AC32,Control	1320.67	v1-x1	132.78	y2-d3	80.30	e3-j3	520.68	i3-l3	0.210	k1-n1	58.44	h1i1	4.65	n2-r2
AC33,Control	839.64	v2-y2	96.98	q3r3	63.82	s3-v3	573.38	u2v2	0.210	k1-n1	136.36	vwx	3.31	o3
AC34,Control	518.36	p3-r3	79.38	v3w3	83.48	d3-f3	563.24	v2w2	0.202	l1-o1	38.96	j1k1	3.61	k3l3
AC35,Control	764.26	a3-e3	156.23	e2-j2	74.12	k3-n3	549.05	x2-a3	0.186	n1-q1	116.88	y-a1	5.23	a2-d2
AC36,Control	778.62	z2-d3	104.38	o3p3	81.99	e3-g3	601.76	k2-o2	0.137	s1-u1	97.40	b1-d1	5.92	m1-o1
AC37,Control	1123.23	g2h2	154.38	f2-k2	62.70	t3-w3	595.68	n2-q2	0.218	j1-m1	71.43	f1-h1	5.21	a2-d2
AC38,Control	825.28	w2-z2	106.23	o3p3	58.58	w3x3	546.35	y2-b3	0.153	r1-t1	58.44	h1i1	5.37	x1-z1
AC39,Control	1403.23	q1-s1	138.95	t2-y2	89.66	a3b3	526.08	f3-j3	0.169	p1-r1	58.44	h1i1	3.95	d3-f3
AC40,Control	936.56	q2-s2	108.70	m3-p3	59.33	v3w3	550.41	x2-a3	0.121	u1	129.87	wxy	4.35	w2-z2
AC41,Control	731.95	d3-g3	95.12	r3s3	54.64	x3y3	529.46	e3-j3	0.161	q1-s1	90.91	c1-e1	4.03	d3e3
AC42,Control	1697.59	g1-i1	214.57	d1-f1	67.57	p3-s3	540.27	a3-d3	0.282	d1-f1	71.43	f1-h1	3.39	n3o3
AC43,Control	620.67	m3-o3	67.96	x3	62.51	t3-w3	519.32	j3-k3	0.178	o1-r1	71.43	f1-h1	3.84	f3-i3
AC44,Control	880.92	t2-v2	176.30	t1-v1	79.93	e3-j3	605.14	i2-n2	0.202	l1-o1	71.43	f1-h1	4.60	p2-s2
AC45,Control	439.38	t3	152.84	h2-l2	76.37	j3-l3	576.76	t2u2	0.194	m1-p1	58.44	h1i1	6.45	a1-c1
AC46,Control	740.92	c3-f3	88.64	t3u3	81.24	e3-i3	535.54	c3-f3	0.153	r1-t1	84.42	d1-f1	5.76	r1s1
AC47,Control	845.03	v2-x2	104.38	o3p3	72.25	l3-o3	470.68	p3	0.274	e1-g1	90.91	c1-e1	3.37	n3o3
AC48,Control	1191.44	d2-f2	166.11	y1-b2	58.39	x3y3	575.41	t2u2	0.323	y-b1	90.91	c1-e1	3.73	i3-k3
AC49,Control	1159.13	e2-g2	160.56	b2-f2	82.36	d3-f3	611.89	f2-k2	0.250	g1-i1	71.43	f1-h1	5.24	a2-d2
AC50,Control	801.95	x2-a3	160.86	a2-e2	105.58	x2	508.51	m3n3	0.331	x-a1	71.43	f1-h1	3.32	n3o3
AC51,Control	796.56	y2-b3	103.15	p3q3	53.33	y3z3	576.08	t2u2	0.194	m1-p1	77.92	e1-g1	4.95	g2-i2
AC52,Control	1406.82	q1-s1	201.30	h1i1	63.82	s3-v3	578.78	s2-u2	0.153	r1-t1	64.94	f1-h1	4.98	f2-h2
AC53,Control	1024.51	k2-o2	75.68	w3	51.09	y3z3	538.24	b3-e3	0.121	u1	77.92	e1-g1	4.27	y2-b3
AC54,Control	496.82	q3-s3	107.16	n3-p3	88.91	a3b3	486.22	o3	0.331	x-a1	77.92	e1-g1	6.37	c1d1
AC55,Control	927.59	r2-t2	123.21	g3-i3	51.46	y3z3	551.08	x2-z2	0.169	p1-r1	77.92	e1-g1	3.94	e3f3
AC56,Control	543.49	p3q3	90.56	s3-u3	80.86	e3-i3	548.38	y2-b3	0.153	r1-t1	116.88	y-a1	4.90	g2-j2
AC57,Control	676.31	i3-l3	77.53	w3	77.12	i3-k3	603.11	i2-o2	0.218	j1-m1	64.94	f1-h1	5.21	a2-d2
AC58,Control	645.79	j3-n3	195.74	i1-m1	59.14	v3w3	472.03	p3	0.169	p1-r1	58.44	h1i1	3.32	n3o3
AC59,Control	819.90	w2-z2	104.69	o3p3	100.15	y2z2	553.78	w2-y2	0.274	e1-g1	149.35	tuv	4.84	i2-l2
AC60,Control	895.28	s2-u2	115.19	j3-l3	50.71	y3z3	461.89	p3	0.194	m1-p1	58.44	h1i1	3.81	g3-i3
AC61,Control	1006.56	n2o2	192.96	j1-n1	88.73	a3b3	522.03	g3-k3	0.218	j1-m1	71.43	f1-h1	5.19	b2-e2
AC62,Control	674.51	i3-l3	77.22	w3	72.06	l3-o3	510.54	l3-n3	0.153	r1-t1	84.42	d1-f1	3.44	m3n3
AC63,Control	1064.00	j2-l2	85.56	u3v3	89.48	a3b3	521.35	h3-k3	0.274	e1-g1	71.43	f1-h1	4.05	d3e3
AC64,Control	1227.33	b2-d2	110.56	l3-o3	88.91	a3b3	530.14	d3-i3	0.202	l1-o1	64.94	f1-h1	4.90	g2-j2
AC1,PEG-7.5	841.44	v2-y2	130.62	a3-f3	225.24	c2d2	697.03	b1-d1	0.420	opq	149.35	tuv	6.19	e1-g1

AC2,PEG-7.5	739.13	c3-f3	167.96	w1-z1	259.70	v1	664.59	n1-u1	0.347	v-y	84.42	d1-f1	4.90	g2-j2
AC3,PEG-7.5	897.08	s2-u2	186.79	n1-q1	234.98	z1a2	717.30	s-v	0.266	f1-h1	142.86	uvw	5.37	x1-z1
AC4,PEG-7.5	1042.46	j2-n2	154.69	e2-k2	398.09	tu	674.05	j1-n1	0.428	nop	175.32	pqr	4.06	c3d3
AC5,PEG-7.5	1295.54	w1-z1	178.15	r1-u1	242.85	x1	663.92	n1-v1	0.339	w-z	142.86	uvw	7.79	hi
AC6,PEG-7.5	631.44	l3-o3	181.54	q1-t1	403.52	s	695.68	c1-f1	0.428	nop	227.27	ij	7.13	pq
AC7,PEG-7.5	638.62	k3-o3	145.43	n2-s2	225.62	c2d2	719.32	r-u	0.371	s-v	149.35	tuv	4.60	p2-s2
AC8,PEG-7.5	794.77	y2-b3	141.11	p2-v2	133.11	s2	642.30	z1-c2	0.186	n1-q1	162.34	rst	6.71	vw
AC9,PEG-7.5	2270.15	u	167.04	x1-a2	312.13	k1l1	722.70	rst	0.347	v-y	279.22	efg	5.31	y1-b2
AC10,PEG-7.5	1205.79	c2-e2	147.28	l2-p2	166.63	n2o2	679.46	h1-k1	0.202	l1-o1	162.34	rst	6.42	b1-d1
AC11,PEG-7.5	617.08	m3-o3	146.36	m2-r2	92.85	a3	736.89	no	0.274	e1-g1	129.87	wxy	5.47	w1x1
AC12,PEG-7.5	1345.79	t1-v1	144.20	o2-t2	214.01	f2	651.08	w1-a2	0.387	rst	162.34	rst	6.71	vw
AC13,PEG-7.5	726.56	e3-h3	136.17	u2-a3	139.10	r2	610.54	f2-l2	0.266	f1-h1	90.91	c1-e1	6.63	wxy
AC14,PEG-7.5	845.03	v2-x2	218.58	c1-e1	198.84	i2	559.19	w2x2	0.315	z-c1	188.31	nop	5.15	d2e2
AC15,PEG-7.5	1991.95	z	230.82	x-b1	214.57	f2	613.85	f2-j2	0.420	opq	194.81	mno	6.47	z-c1
AC16,PEG-7.5	1067.59	j2-k2	181.23	q1-t1	185.54	k2l2	584.86	r2-t2	0.379	stu	188.31	nop	5.55	v1w1
AC17,PEG-7.5	1701.18	g1-i1	189.57	m1-o1	220.37	e2	636.89	b2-d2	0.323	y-b1	188.31	nop	5.84	n1-r1
AC18,PEG-7.5	1668.87	h1-j1	125.99	e3-h3	170.75	m2n2	688.92	d1-h1	0.282	d1-f1	149.35	tuv	5.23	a2-d2
AC19,PEG-7.5	1376.31	s1-u1	131.23	z2-e3	154.83	p2	654.46	u1-x1	0.218	j1-m1	188.31	nop	6.21	e1f1
AC20,PEG-7.5	1019.13	l2-o2	195.74	i1-m1	164.01	o2	645.00	x1-b2	0.282	d1-f1	162.34	rst	5.27	z1-c2
AC21,PEG-7.5	1304.51	v1-x1	244.51	uv	204.46	g2h2	697.03	b1-d1	0.307	a1-d1	188.31	nop	5.32	y1-a2
AC22,PEG-7.5	1711.95	f1-h1	219.20	c1-e1	194.16	j2	706.49	w-b1	0.323	y-b1	168.83	qrs	6.06	h1-k1
AC23,PEG-7.5	1430.15	q1r1	219.51	c1-e1	201.09	h2i2	677.43	i1-m1	0.428	nop	175.32	pqr	7.31	n
AC24,PEG-7.5	1317.08	v1-x1	233.09	x-a1	248.46	w1	738.92	mno	0.323	y-b1	214.29	jkl	5.73	r1-s1
AC25,PEG-7.5	1157.33	f2g2	241.42	vw	220.75	e2	662.57	o1-v1	0.371	s-v	201.30	lmn	6.90	tu
AC26,PEG-7.5	2462.21	rs	305.93	gh	332.73	g1h1	684.86	g1-i1	0.379	stu	233.77	hi	5.92	m1-o1
AC27,PEG-7.5	2205.54	v	258.09	pqr	366.25	a1	665.27	n1-t1	0.476	kl	220.78	ijk	3.77	h3-j3
AC28,PEG-7.5	2521.44	pq	276.60	l	290.22	o1p1	678.11	i1-l1	0.395	qrs	220.78	ijk	7.16	op
AC29,PEG-7.5	1119.64	g2-i2	227.84	z-b1	272.43	s1	668.65	l1-q1	0.307	a1-d1	188.31	nop	7.47	lm
AC30,PEG-7.5	751.69	b3-e3	166.11	y1-b2	149.78	q2	684.86	g1-i1	0.339	w-z	149.35	tuv	6.66	wx
AC31,PEG-7.5	1611.44	k1l1	196.36	i1-l1	339.10	e1f1	641.62	z1-d2	0.363	t-w	214.29	jkl	5.18	c2-e2
AC32,PEG-7.5	1918.36	a1	229.69	y-b1	358.39	b1	631.49	d2e2	0.420	opq	201.30	lmn	5.29	y1-c2
AC33,PEG-7.5	1011.95	m2-o2	126.30	e3-h3	183.67	l2	659.86	p1-w1	0.282	d1-f1	214.29	jkl	5.45	w1x1
AC34,PEG-7.5	1738.87	d1-g1	156.85	d2-j2	311.01	k1l1	656.49	r1-w1	0.444	mno	175.32	pqr	4.60	p2-s2
AC35,PEG-7.5	1187.85	d2-f2	146.98	l2-q2	171.31	m2	643.65	y1-b2	0.266	f1-h1	155.84	stu	6.95	st
AC36,PEG-7.5	1317.08	v1-x1	134.01	x2-d3	219.06	e2	666.62	n1-r1	0.299	b1-e1	162.34	rst	7.10	pqr
AC37,PEG-7.5	1209.38	c2-d2	200.68	h1i1	149.78	q2	697.03	b1-d1	0.331	x-a1	149.35	tuv	7.08	pqr
AC38,PEG-7.5	2072.72	y	204.38	g1h1	336.67	f1g1	640.95	a2-d2	0.355	u-x	188.31	nop	5.63	t1-v1
AC39,PEG-7.5	2643.49	n	309.94	fg	370.94	z	698.38	a1-d1	0.387	rst	188.31	nop	4.76	k2-n2
AC40,PEG-7.5	1381.69	s1-u1	149.14	k2-o2	237.98	y1z1	659.19	q1-w1	0.379	stu	149.35	tuv	5.82	o1-r1
AC41,PEG-7.5	1412.21	q1-s1	191.73	k1-n1	326.93	i1	655.81	s1-w1	0.363	t-w	149.35	tuv	4.79	j2-m2
AC42,PEG-7.5	2458.62	rs	233.40	xyz	395.84	u	680.14	h1-k1	0.452	lmn	181.82	opq	3.90	f3g3
AC43,PEG-7.5	1414.00	q1-s1	192.96	j1-n1	421.87	q	688.92	d1-h1	0.290	c1-f1	168.83	qrs	4.52	s2-v2
AC44,PEG-7.5	1040.67	j2-n2	214.57	d1-f1	205.77	g2	664.59	n1-u1	0.387	rst	110.39	z-b1	5.55	v1w1
AC45,PEG-7.5	1078.36	h2-j2	248.21	tu	249.96	w1	684.19	h1-j1	0.282	d1-f1	97.40	b1-d1	7.97	fg
AC46,PEG-7.5	1699.38	g1-i1	177.84	r1-u1	379.74	wx	671.35	k1-o1	0.347	v-y	162.34	rst	6.11	f1-i1
AC47,PEG-7.5	2108.62	wxy	141.73	p2-u2	396.03	u	616.62	f2-h2	0.411	pqr	168.83	qrs	3.87	f3-h3
AC48,PEG-7.5	1819.64	b1c1	235.56	wxy	259.14	v1	671.35	k1-o1	0.379	stu	136.36	vwx	4.45	t2-w2
AC49,PEG-7.5	1444.51	p1q1	159.01	c2-h2	183.11	l2	670.00	k1-p1	0.355	u-x	162.34	rst	7.03	qrs
AC50,PEG-7.5	1842.97	b1	204.69	g1h1	282.73	q1	588.24	q2-s2	0.508	ij	168.83	qrs	4.42	v2-x2
AC51,PEG-7.5	1245.28	a2-c2	167.35	w1-z1	229.55	b2c2	683.51	h1-j1	0.323	y-b1	162.34	rst	7.00	rst
AC52,PEG-7.5	1539.64	n1	189.88	m1-o1	241.54	x1y1	710.54	u-z	0.379	stu	123.38	xyz	6.13	f1-h1
AC53,PEG-7.5	1537.85	n1	150.68	j2-n2	265.51	t1u1	719.32	r-u	0.299	b1-e1	136.36	vwx	5.28	y1-c2
AC54,PEG-7.5	1776.56	c1d1	213.95	e1f1	431.24	p	611.89	f2-k2	0.500	jk	207.79	klm	7.08	pqr
AC55,PEG-7.5	1399.64	q1-s1	141.11	p2-v2	400.71	st	703.78	y-c1	0.347	v-y	162.34	rst	4.92	g2-i2
AC56,PEG-7.5	1602.46	l1	180.31	r1-t1	341.91	d1e1	667.30	m1-q1	0.339	w-z	175.32	pqr	5.63	t1-v1
AC57,PEG-7.5	929.38	r2s2	124.75	f3-i3	230.30	b2	682.84	h1-j1	0.331	x-a1	194.81	mno	7.11	pqr
AC58,PEG-7.5	954.51	p2-r2	339.88	e	345.09	d1	534.19	c3-f3	0.323	y-b1	175.32	pqr	4.76	k2-n2
AC59,PEG-7.5	1072.97	i2j2	163.95	z1-c2	233.48	a2b2	651.76	w1-z1	0.363	t-w	272.73	fg	6.31	d1e1
AC60,PEG-7.5	1042.46	j2-n2	158.09	c2-i2	340.41	e1f1	615.27	f2-i2	0.331	x-a1	155.84	stu	4.68	m2-q2
AC61,PEG-7.5	2961.18	l	264.57	no	505.58	j	660.54	p1-w1	0.363	t-w	305.19	bc	5.84	n1-r1
AC62,PEG-7.5	1279.38	x1-a1	176.91	s1-v1	289.66	o1p1	640.95	a2-d2	0.307	a1-d1	162.34	rst	4.55	r2-u2
AC63,PEG-7.5	2582.46	o	148.52	k2-o2	453.15	n	651.08	w1-a2	0.420	opq	181.82	opq	4.63	o2-s2
AC64,PEG-7.5	1597.08	l1	184.01	o1-r1	301.46	n1	620.68	f2g2	0.347	v-y	149.35	tuv	5.98	j1-1
AC1,PEG-15	1340.41	u1-w1	201.30	h1i1	317.75	j1	696.35	c1-e1	0.331	x-a1	136.36	vwx	8.13	cd
AC2,PEG-15	952.72	p2-r2	226.91	a1b1	374.49	yz	686.22	e1-i1	0.266	f1-h1	97.40	b1-d1	5.69	s1-u1
AC3,PEG-15	1058.62	j2-m2	203.46	g1h1	341.16	d1e1	741.62	k-n	0.250	g1-i1	116.88	y-a1	6.16	f1-h1
AC4,PEG-15	3386.56	i	311.17	fg	514.76	i	791.62	e	0.541	fgh	311.69	b	4.31	x2-a3
AC5,PEG-15	1765.79	d1e1	301.60	hi	352.21	c1	686.22	e1-i1	0.363	t-w	110.39	z-b1	9.19	a

AC6,PEG-15	3241.18	k	271.36	lm	542.28	g	839.59	a	0.589	bcd	266.23	g	6.21	e1f1
AC7,PEG-15	1604.26	l1	172.90	u1-x1	380.67	wx	767.97	gh	0.371	s-v	181.82	opq	4.95	g2-i2
AC8,PEG-15	904.26	s2-u2	192.04	k1-n1	225.24	c2d2	632.16	c2d2	0.234	i1-k1	155.84	stu	8.40	b
AC9,PEG-15	4607.08	b	286.79	k	553.33	f	834.86	ab	0.581	cde	77.92	e1-g1	5.00	f2g2
AC10,PEG-15	1755.03	d1-f1	298.52	ij	327.12	i1	736.89	no	0.282	d1-f1	149.35	tuv	7.85	gh
AC11,PEG-15	841.44	v2-y2	129.69	b3-f3	189.10	k2	734.86	n-q	0.234	i1-k1	84.42	d1-f1	6.10	f1-j1
AC12,PEG-15	2271.95	u	385.56	b	364.76	a1	754.46	i	0.452	lmn	175.32	pqr	7.13	pq
AC13,PEG-15	857.59	u2-w2	152.53	i2-m2	210.26	f2	611.89	f2-k2	0.242	h1-j1	71.43	f1-h1	8.00	ef
AC14,PEG-15	1234.51	a2-d2	156.23	e2-j2	262.13	u1v1	570.68	u2v2	0.282	d1-f1	149.35	tuv	6.13	f1-h1
AC15,PEG-15	2914.51	lm	266.11	mn	373.75	yz	712.57	t-y	0.323	y-b1	155.84	stu	7.15	opq
AC16,PEG-15	1548.62	m1n1	196.67	i1-k1	289.10	p1	665.27	n1-t1	0.347	v-y	149.35	tuv	6.53	y-b1
AC17,PEG-15	2246.82	uv	208.70	f1g1	334.23	g1h1	655.14	t1-x1	0.379	stu	136.36	vwx	6.05	h1-l1
AC18,PEG-15	2126.56	wx	130.93	z2-f3	293.97	o1	686.22	e1-i1	0.323	y-b1	136.36	vwx	6.06	h1-k1
AC19,PEG-15	1914.77	a1	96.36	r3s3	240.04	x1y1	660.54	p1-w1	0.218	j1-m1	181.82	opq	7.18	op
AC20,PEG-15	1266.82	y1-b2	134.63	w2-c3	266.63	t1	654.46	u1-x1	0.290	c1-f1	116.88	y-a1	6.08	g1-j1
AC21,PEG-15	1658.10	i1-k1	217.65	d1e1	275.24	r1s1	716.62	s-w	0.234	i1-k1	181.82	opq	5.89	m1-q1
AC22,PEG-15	2124.77	wx	236.79	wx	279.36	q1r1	735.54	no	0.387	rst	123.38	xyz	6.37	c1d1
AC23,PEG-15	2361.69	t	220.43	c1d1	308.58	l1m1	736.89	no	0.524	hij	168.83	qrs	7.69	ij
AC24,PEG-15	2148.10	w	251.91	rst	380.86	w	725.41	p-s	0.379	stu	188.31	nop	6.05	h1-l1
AC25,PEG-15	1593.49	l1m1	209.32	f1g1	279.36	q1r1	697.70	b1-d1	0.331	x-a1	194.81	mno	7.26	no
AC26,PEG-15	3783.23	g	393.27	a	486.10	k	774.73	fg	0.524	hij	292.21	cde	6.11	f1-i1
AC27,PEG-15	4714.77	a	396.36	a	512.13	i	793.65	e	0.605	bc	305.19	bc	4.18	b3c3
AC28,PEG-15	3379.38	i	299.14	ij	481.24	l	815.95	cd	0.468	lm	279.22	efg	6.45	a1-c1
AC29,PEG-15	1719.13	e1-g1	250.68	stu	390.79	v	778.78	f	0.444	mno	188.31	nop	7.52	kl
AC30,PEG-15	520.15	p3-r3	92.35	r3-t3	273.00	s1	664.59	n1-u1	0.339	w-z	116.88	y-a1	7.35	mn
AC31,PEG-15	3395.54	i	275.99	l	477.68	l	710.54	u-z	0.476	kl	292.21	cde	5.21	a2-d2
AC32,PEG-15	3323.74	j	313.33	f	459.70	m	758.51	hi	0.500	jk	285.71	def	5.60	u1v1
AC33,PEG-15	807.33	x2-a3	120.12	h3-j3	279.18	q1r1	685.54	f1-i1	0.274	e1-g1	149.35	tuv	6.00	i1-m1
AC34,PEG-15	2433.49	s	230.00	y-b1	414.76	r	770.68	fg	0.524	hij	201.30	lmn	5.55	v1w1
AC35,PEG-15	985.03	o2p2	153.15	h2-l2	288.73	p1	665.95	n1-s1	0.250	g1-i1	136.36	vwx	8.10	de
AC36,PEG-15	1478.62	o1p1	162.72	z1-d2	305.77	m1n1	753.11	ij	0.242	h1-j1	168.83	qrs	8.24	c
AC37,PEG-15	1252.46	z1-c2	164.26	z1-c2	222.06	d2e2	689.59	d1-h1	0.315	z-c1	123.38	xyz	8.47	b
AC38,PEG-15	3716.82	h	266.11	mn	504.08	j	724.73	qrs	0.444	mno	233.77	hi	5.92	m1-o1
AC39,PEG-15	4312.72	c	293.27	j	563.45	d	825.41	bc	0.500	jk	292.21	cde	5.13	d2e2
AC40,PEG-15	1624.00	j1-l1	134.94	v2-b3	314.01	j1k1	700.41	z-c1	0.339	w-z	136.36	vwx	6.53	y-b1
AC41,PEG-15	2279.13	u	152.22	i2-m2	443.22	o	748.38	i-m	0.468	lm	201.30	lmn	5.53	v1w1
AC42,PEG-15	4021.95	e	303.15	hi	558.76	e	807.84	d	0.557	efg	246.75	h	4.55	r2-u2
AC43,PEG-15	3352.46	ij	253.15	q-t	575.06	c	826.08	bc	0.533	ghi	227.27	ij	4.87	h2-k2
AC44,PEG-15	1510.92	n1o1	214.26	d1-f1	330.30	h1i1	695.00	c1-h1	0.307	a1-d1	103.90	a1-c1	6.60	wxy
AC45,PEG-15	2329.38	t	199.75	h1i1	402.96	s	754.46	i	0.379	stu	90.91	c1-e1	8.16	cd
AC46,PEG-15	2485.54	qr	255.62	qrs	522.62	h	795.00	e	0.452	lmn	207.79	klm	6.31	d1e1
AC47,PEG-15	3377.59	i	180.31	r1-t1	544.72	g	715.27	s-x	0.565	def	214.29	jkl	4.74	l2-o2
AC48,PEG-15	2088.87	xy	195.43	i1-m1	376.37	xy	771.35	fg	0.428	nop	103.90	a1-c1	5.77	q1-s1
AC49,PEG-15	1128.62	g2	171.36	v1-y1	302.40	n1	698.38	a1-d1	0.347	v-y	90.91	c1-e1	6.56	x-a1
AC50,PEG-15	3207.08	k	264.26	nop	490.22	k	725.41	p-s	0.613	b	214.29	jkl	4.56	q2-t2
AC51,PEG-15	1349.38	t1-v1	176.60	t1-v1	357.27	b1	776.76	fg	0.242	h1-j1	129.87	wxy	7.92	fg
AC52,PEG-15	1905.79	a1	224.75	b1c1	349.78	c1	742.97	j-n	0.339	w-z	103.90	a1-c1	6.58	xyz
AC53,PEG-15	2672.21	n	199.14	h1-j1	422.81	q	811.22	d	0.444	mno	168.83	qrs	5.69	s1-u1
AC54,PEG-15	3971.69	f	258.40	opq	590.60	b	728.78	o-r	0.654	a	298.70	bcd	7.31	n
AC55,PEG-15	2889.38	m	188.95	n1-p1	510.64	i	771.35	fg	0.452	lmn	214.29	jkl	5.84	n1-r1
AC56,PEG-15	3237.59	k	115.19	j3-l3	390.97	v	751.76	ijk	0.452	lmn	214.29	jkl	6.39	c1d1
AC57,PEG-15	595.54	o3	84.32	u3v3	351.65	c1	708.51	v-a1	0.339	w-z	123.38	xyz	7.63	jk
AC58,PEG-15	2431.69	s	355.93	d	397.15	tu	655.14	t1-x1	0.452	lmn	168.83	qrs	5.90	m1-p1
AC59,PEG-15	1543.23	n1	139.57	s2-x2	342.28	d1e1	697.70	b1-d1	0.355	u-x	181.82	opq	7.45	lm
AC60,PEG-15	1756.82	d1-f1	224.75	b1c1	435.54	p	705.14	x-c1	0.371	s-v	103.90	a1-c1	5.55	v1w1
AC61,PEG-15	4289.38	c	374.14	c	617.19	a	833.51	ab	0.589	bcd	350.65	a	5.79	p1-s1
AC62,PEG-15	2672.21	n	188.95	n1-p1	402.58	s	740.95	lmn	0.395	qrs	188.31	nop	5.39	x1-z1
AC63,PEG-15	4100.92	d	250.99	st	591.35	b	810.54	d	0.524	hij	285.71	def	4.97	f2-h2
AC64,PEG-15	2566.31	op	159.94	b2-g2	395.84	u	751.08	i-l	0.395	qrs	188.31	nop	6.89	tu

PC: proline content, SSC: soluble sugar content, TPC: total phenolic content, AC: antioxidant activity, GPA: guaiacol peroxidase activity, CAT: catalase activity, LP: lipid peroxidation. Different letters denote a statistically significant variation in mean values measured by Duncan's Multiple-Range Test ($p \leq 0.01$).
